# Metaheuristic hyperparameter optimization of deep neural networks for demographic-aware autism spectrum disorder classification

**DOI:** 10.1038/s41598-026-58789-0

**Published:** 2026-06-29

**Authors:** Mohammed Aly, Naif M. Alotaibi

**Affiliations:** 1 Department of Artificial Intelligence, Faculty of Artificial Intelligence, Egyptian Russian University, Badr City, 11829 Egypt; 2 Department of Software Engineering, Faculty of Artificial Intelligence, Egyptian Russian University, Badr City, 11829 Egypt; 3 Department of Computer Science, College of Science and Humanities Dawadmi, Shaqra University, Shaqra, 11961 Saudi Arabia

**Keywords:** Deep learning, Metaheuristic optimization, Artificial bee colony, Convolutional neural networks, Multi-class classification, Autism spectrum disorder, Computational biology and bioinformatics, Engineering, Health care, Mathematics and computing

## Abstract

Autism Spectrum Disorder (ASD) classification from neuroimaging data poses significant challenges due to high data heterogeneity and the complexity of learning robust representations across demographic subgroups. While recent deep learning approaches have shown promise, most existing studies rely on binary classification and limited model tuning strategies, overlooking demographic variability and the role of systematic optimization in model design. To address these challenges, this study formulates demographic-aware ASD classification as an optimization-driven learning problem and proposes a deep learning framework based on structural MRI (sMRI) data. The proposed framework employs three customized Convolutional Neural Network (CNN) models targeting gender-based classification, age-group-based classification, and joint age–gender classification using an octal class structure. Model architecture and training hyperparameters are automatically optimized using the Optimized Artificial Bee Colony (OptABC) algorithm, enabling task-specific adaptation without manual tuning. A dedicated preprocessing pipeline incorporating structural localization and controlled data augmentation is applied to improve robustness across heterogeneous imaging sites. All models are evaluated using five-fold cross-validation on the multi-site ABIDE dataset. Experimental results demonstrate that the proposed optimization-driven framework achieves accuracies of 84.25%, 88.07%, and 71.58% for gender-based, age-based, and joint age–gender classification tasks, respectively, yielding competitive performance relative to widely used pre-trained transfer learning models under comparable experimental settings. The results further indicate that age-aware modeling offers stronger discriminative capability than gender-based classification, while joint demographic stratification introduces increased task complexity due to finer class granularity. Overall, the findings highlight the effectiveness of metaheuristic optimization for enhancing deep learning models in complex, demographic-aware neuroimaging classification tasks. Further evaluation on independent datasets is planned to assess robustness across broader settings.

**Supplementary Information:**

The online version contains supplementary material available at 10.1038/s41598-026-58789-0.

## Introduction

Autism Spectrum Disorder (ASD) is a complex neurodevelopmental condition characterized by substantial heterogeneity in clinical presentation, posing significant challenges for conventional diagnostic approaches. This variability necessitates advanced methodologies that not only improve diagnostic accuracy but also account for demographic factors such as age and gender. Recent advances in neuroimaging, particularly Magnetic Resonance Imaging (MRI), have enabled detailed investigation of brain structure, providing valuable insights into ASD-related neuroanatomical alterations^1,2^.

To address the limitations of traditional diagnostic methods, this study introduces a deep learning (DL)-based framework for ASD classification. As a subset of artificial intelligence, DL has demonstrated strong capability in learning discriminative representations from high-dimensional medical imaging data. In this work, these capabilities are leveraged to identify subtle structural patterns in brain MRI scans associated with ASD, while explicitly modeling the influence of age and gender on disease manifestation.

ASD is commonly associated with impairments in social communication and the presence of repetitive behaviors, creating challenges for both clinicians and researchers. Although prior neuroimaging studies have reported structural brain abnormalities in individuals with ASD, the integration of deep learning techniques for ASD classification remains an evolving research direction. By incorporating age and gender into the learning process, this study moves beyond conventional one-size-fits-all models and acknowledges the diverse developmental trajectories and phenotypic expressions of ASD across demographic groups^3^.

The importance of age-aware modeling in ASD diagnosis is emphasized by the dynamic nature of neurodevelopment. Brain structure undergoes continuous changes across the lifespan, and age-specific patterns may provide valuable diagnostic cues. Similarly, incorporating gender as a classification factor reflects well-documented differences in ASD prevalence and symptom expression, contributing to a more comprehensive understanding of the disorder^4^.

The performance and design of deep learning models for ASD diagnosis are strongly influenced by dataset characteristics, including demographic diversity, imaging protocols, and data acquisition sites. Such variability presents a major challenge in developing robust and generalizable diagnostic systems. Addressing this challenge requires careful architectural design, demographic stratification, and effective preprocessing strategies, including normalization, data augmentation, and quality control. Furthermore, appropriate evaluation metrics and validation across heterogeneous datasets are essential for assessing real-world applicability. Continuous model refinement, supported by clinical insight and multi-site validation, is therefore critical for improving diagnostic reliability in ASD^5–7^.

In this work, we employ state-of-the-art deep learning techniques to analyze a large-scale brain MRI dataset comprising individuals diagnosed with ASD and typically developing controls. The primary objective is to enhance diagnostic precision within a personalized medicine framework by integrating demographic information into the classification process. By jointly modeling age and gender, the proposed approach not only improves diagnostic performance but also provides insight into demographic-specific neuroanatomical patterns associated with ASD.

This study systematically investigates the influence of age and gender through a structured, demographic-aware multi-class learning formulation for Autism Spectrum Disorder (ASD) classification. In contrast to most existing studies that primarily address ASD as a binary classification problem, the proposed framework explicitly models demographic stratification by performing age-based, gender-based, and joint age–gender classification. To the best of our knowledge, this work represents one of the first attempts to jointly examine age- and gender-aware multi-class ASD classification within an optimization-driven deep learning framework. By leveraging a large, heterogeneous, multi-site neuroimaging dataset, the proposed approach is evaluated under realistic and diverse acquisition conditions. A convolutional neural network (CNN) architecture is designed and trained from scratch to enable automatic feature learning, and its performance is systematically benchmarked against widely used pre-trained transfer learning models under identical experimental settings.

The main contributions of this study are summarized as follows:

1. The formulation of a demographic-aware, multi-class ASD classification framework that extends beyond conventional binary learning paradigms;
2. The development of a structured preprocessing pipeline incorporating edge-based feature enhancement and controlled data augmentation;
3. The integration of metaheuristic hyperparameter optimization using the Optimized Artificial Bee Colony (OptABC) algorithm to improve learning efficiency and generalization;
4. The design of three task-specific CNN models tailored to age-based, gender-based, and joint demographic stratification;
5. Extensive experimental validation using five-fold cross-validation on the multi-site ABIDE dataset, demonstrating the effectiveness and scalability of the proposed optimization-driven approach.

The remainder of the paper is organized as follows. Section 2 reviews related work on ASD classification using brain MRI with emphasis on demographic factors. Section 3 describes the dataset, preprocessing steps, and proposed methodology. Section 4 presents the evaluation metrics, while Sect. 5 discusses the experimental results and analysis. Finally, Sect. 6 concludes the paper and outlines directions for future research.

## Related work

Autism Spectrum Disorder (ASD) comprises a group of neurodevelopmental conditions characterized by persistent deficits in social interaction and communication, along with restricted and repetitive patterns of behavior. Conventional ASD diagnosis relies primarily on behavioral assessments, which can be subjective and time-consuming. In recent years, magnetic resonance imaging (MRI), combined with machine learning (ML) and deep learning (DL) techniques, has emerged as a promising avenue for developing objective and data-driven diagnostic tools. Both functional MRI (fMRI) and structural MRI (sMRI) have been widely explored, with DL models demonstrating strong potential for capturing complex neuroanatomical and functional patterns associated with ASD.

Early neuroimaging-based ASD classification studies primarily employed handcrafted features followed by traditional classifiers. More recent work has shifted toward deep architectures capable of learning hierarchical representations directly from imaging data. Maryam et al.^8^ proposed a deep belief network (DBN) that integrated multimodal data from ABIDE I and ABIDE II, combining rs-fMRI with gray- and white-matter features derived from the AAL atlas. Their approach demonstrated the effectiveness of deep feature fusion for early ASD diagnosis, particularly in pediatric cohorts. However, the framework focused on binary classification and did not explore demographic stratification or multi-class diagnostic scenarios.

Similarly, Mohamed T. et al.^9^ investigated structural abnormalities associated with ASD using cortical and volumetric morphological features extracted via FreeSurfer. By employing recursive feature selection and conventional classifiers, their method achieved reasonable classification performance, highlighting the relevance of structural biomarkers. Nevertheless, reliance on handcrafted features and binary classification limits scalability and the ability to capture complex demographic-dependent patterns.

Despite the growing body of work on MRI-based ASD classification, most studies continue to emphasize binary ASD versus typically developing (TD) discrimination. This focus restricts the capacity of models to reflect the intrinsic heterogeneity of ASD across demographic subgroups. In contrast, demographic-aware modeling has recently gained attention as a means of capturing age- and gender-specific neurodevelopmental trajectories. Vigneshwaran S. et al.^10^ introduced an enhanced radial basis function neural classifier (EMcRBFN) and demonstrated improved performance on ABIDE data while identifying age- and gender-specific brain regions associated with ASD. Although this study highlights the importance of demographic stratification, it relies on shallow architectures and does not exploit end-to-end deep learning for feature extraction.

Advanced DL-based frameworks have also explored connectivity-driven and adversarial learning strategies. Yazhou K. et al.^11^ constructed individual brain networks from T1-weighted MRI and trained a deep neural network using discriminative connectivity features, achieving high precision and AUC. Yan T. et al.^12^ proposed a two-phase adversarial learning model with a sliding-window mechanism to mitigate multi-site variability in rs-fMRI data, demonstrating improved robustness to site heterogeneity. While these methods address specific challenges such as connectivity modeling and inter-site variability, they remain primarily focused on binary classification and often involve complex preprocessing pipelines.

Beyond imaging-based approaches, Dhuha D. et al.^13^ developed age-stratified ML models for ASD prediction using questionnaire-based screening datasets. Although near-perfect accuracies were reported, the non-imaging nature of the data limits comparability with MRI-based diagnostic frameworks. Suckling J. et al.^14^ addressed a four-class classification problem based on sex and diagnosis using voxel-based morphometry features and partial least squares modeling. Their work demonstrates the feasibility of demographic-aware multi-class neuroimaging analysis but relies on classical feature extraction rather than fully end-to-end DL architectures.

More recently, high-quality studies have introduced hybrid and optimization-driven frameworks for ASD diagnosis. Khan and Katarya^15^ proposed MCBERT, a multi-modal framework that integrates structural and functional MRI using transformer-based representations, achieving improved diagnostic performance. While effective, the model primarily focuses on overall ASD classification without explicitly modeling demographic heterogeneity. In another work, Khan and Katarya^16^ introduced AFF-BPL, an adaptive feature fusion framework combining Bat-PSO optimization with LSTM architectures. This approach highlights the benefit of hybrid optimization and feature fusion but remains limited to binary classification and does not directly address age- or gender-specific variability. Similarly, WS-BiTM integrates White Shark Optimization with Bi-LSTM networks to enhance ASD diagnosis^17^, demonstrating the value of advanced metaheuristic optimization while still emphasizing performance improvement rather than demographic-aware analysis.

In addition to earlier deep learning approaches, several recent studies published between 2023 and 2025 have further advanced ASD diagnosis through diverse data modalities, optimization strategies, and learning paradigms. Khan and Katarya proposed MCBERT, a transformer-based multi-modal framework that integrates structural and functional neuroimaging representations, demonstrating improved diagnostic capability through cross-modal attention mechanisms^15^. While effective, the framework primarily focuses on overall ASD classification without explicit demographic stratification.

More recently, WS-BiTM integrates White Shark Optimization with Bi-LSTM architectures to enhance feature learning and convergence stability in ASD diagnosis^16^. This work highlights the benefit of metaheuristic optimization but remains centered on binary classification objectives. Similarly, AFF-BPL introduces an adaptive feature fusion strategy combining Bat-PSO optimization with LSTM models, achieving improved performance through hybrid optimization while still relying on feature-level fusion rather than end-to-end demographic-aware learning^17^.

Complementary perspectives are provided by survey and empirical studies that analyze broader methodological trends. For example, Khan and Katarya^18^ reviewed prevailing machine learning techniques for ASD diagnosis and emphasized challenges related to generalizability, interpretability, and dataset heterogeneity. Other studies have explored alternative deep architectures, such as Xception-based neuroimaging models^19^, and conducted empirical evaluations of classical and deep learning classifiers for ASD prediction^20^. Beyond neuroimaging, recent work has also investigated self-supervised and self-distillation learning paradigms for ASD classification using facial images, illustrating the growing interest in scalable and data-efficient learning strategies^21^.

Collectively, these recent studies underscore a shift toward more advanced optimization and learning mechanisms, while also revealing persistent gaps in demographic-aware modeling and multi-class neuroimaging-based analysis. These limitations motivate the present study, which explicitly integrates age and gender stratification within an end-to-end CNN framework optimized for structural MRI data.

Collectively, these studies reveal several persistent limitations in the existing literature. First, the predominance of binary classification paradigms limits the ability to capture ASD heterogeneity across age and gender. Second, many approaches either rely on handcrafted features or employ complex hybrid pipelines that hinder interpretability and practical deployment. Third, although optimization and multi-modal strategies improve accuracy, demographic stratification is rarely integrated directly into the learning objective, and multi-class formulations remain underexplored.

Motivated by these gaps, the present study proposes a demographic-aware, multi-class CNN framework optimized using a metaheuristic algorithm. By explicitly modeling age-based, gender-based, and joint age–gender classification scenarios using sMRI data, the proposed approach extends beyond conventional binary diagnosis and provides a more granular representation of ASD-related neuroanatomical variations. Furthermore, through rigorous cross-validation and extended analytical evaluation, this work aims to balance classification performance, model complexity, and practical applicability, addressing key limitations identified in prior studies.

## Materials and proposed method

### Dataset

This study is conducted using data from the Autism Brain Imaging Data Exchange (ABIDE), a large-scale, multi-site, open-access neuroimaging repository designed to support reproducible research on Autism Spectrum Disorder (ASD). ABIDE aggregates data from 37 international research sites, incorporating variability in scanners, acquisition protocols, and demographic characteristics, thereby providing a realistic benchmark for evaluating data-driven diagnostic models^22^.

ABIDE offers multiple neuroimaging modalities, including structural MRI (sMRI), resting-state fMRI, and diffusion-weighted imaging. In this study, only T1-weighted sMRI scans are utilized. In addition, the dataset provides rich demographic metadata, including age, gender, and diagnostic status, enabling stratified analysis. Standardized preprocessing pipelines supplied with ABIDE include motion correction, spatial normalization, and noise reduction, which further support cross-site consistency^23^.

After automated quality control and visual inspection, low-quality or unclear scans were excluded. From the original dataset, a final cohort of 1,645 subjects was retained, comprising 798 individuals diagnosed with ASD and 847 typically developing (TD) controls, with ages ranging from 6.3 to 40 years.

**Fig. 1:**
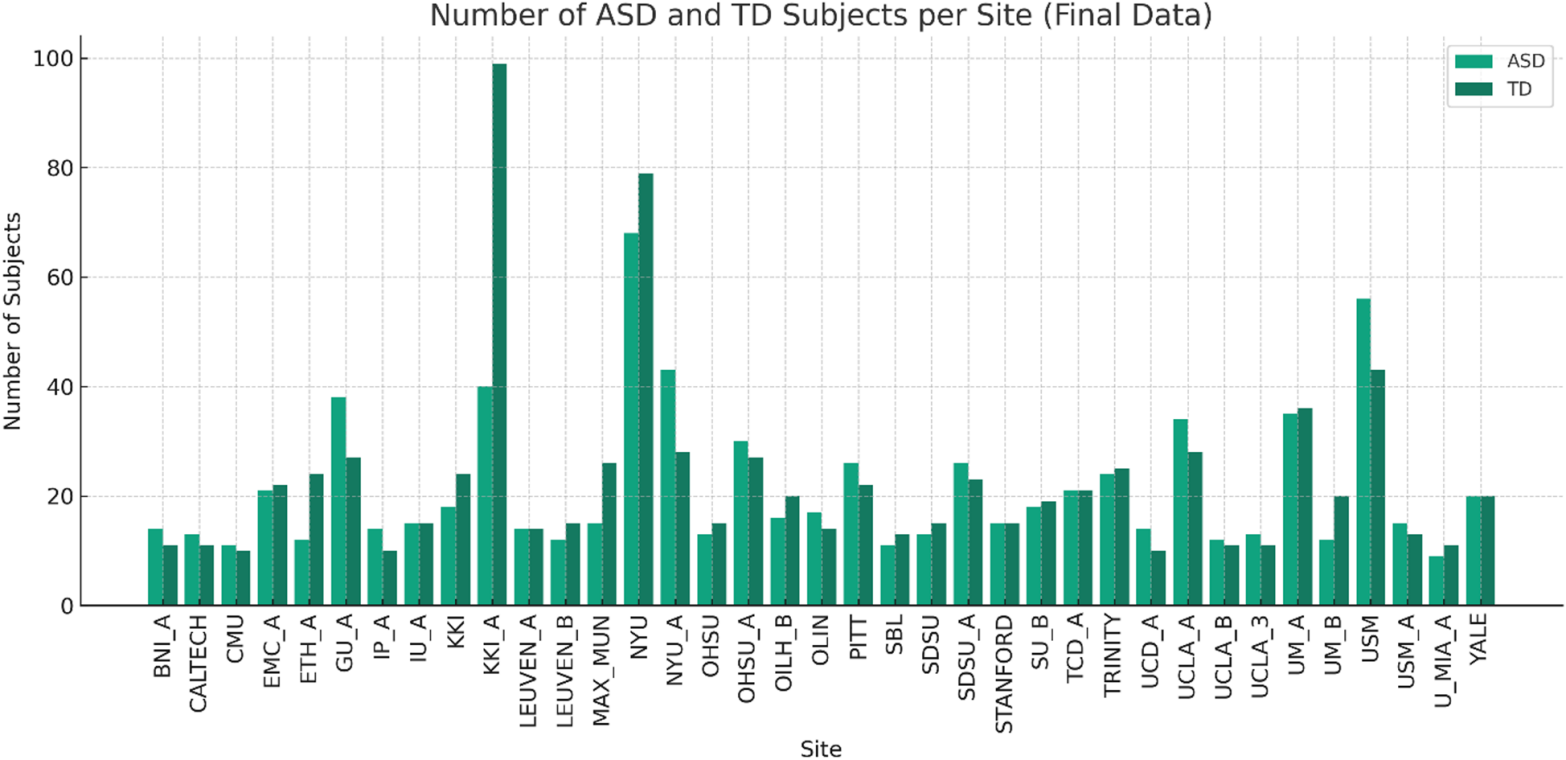
Site-wise distribution of ASD and typically developing (TD) participants in the final cleaned ABIDE dataset. The figure illustrates variability in sample size across acquisition sites, emphasizing the heterogeneity addressed by the proposed framework.

**Fig. 2:**
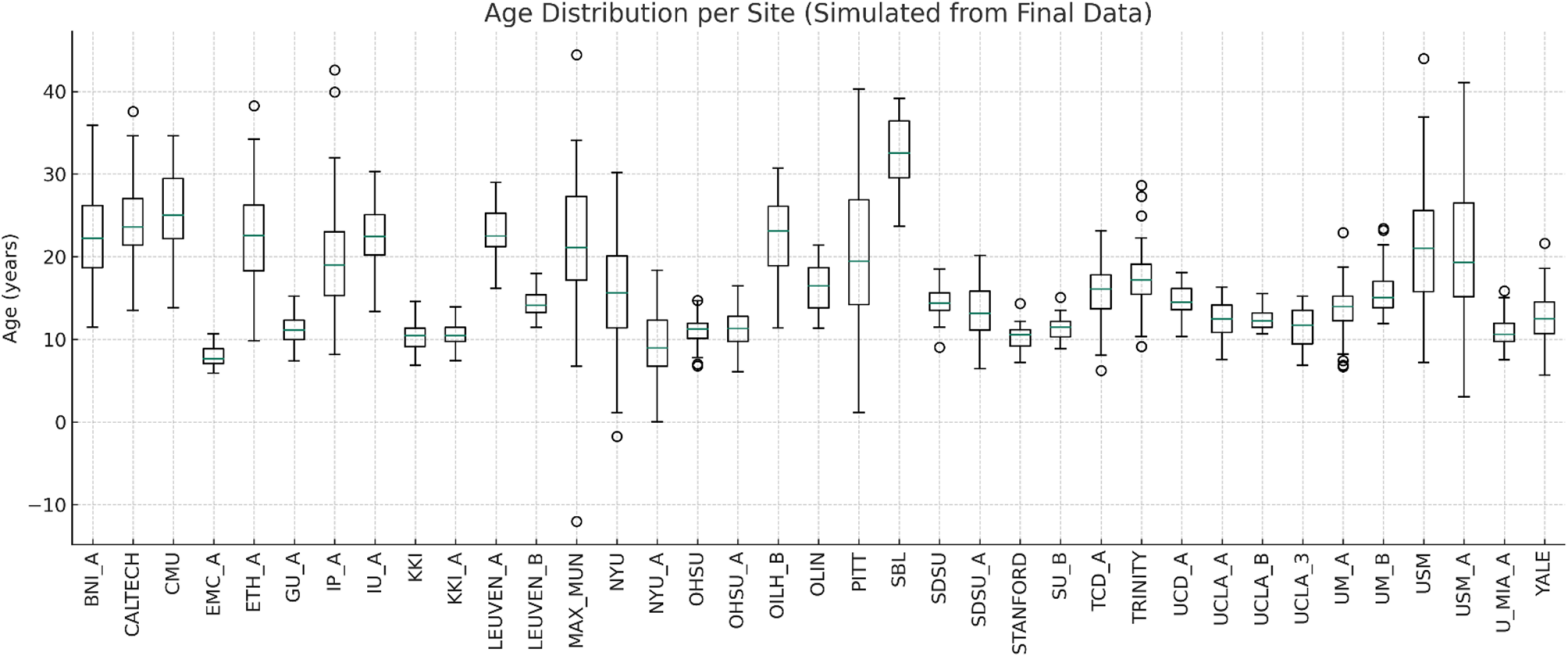
Boxplot representation of age distributions across ABIDE acquisition sites for ASD and TD participants. The figure highlights age variability within and across sites, motivating the need for age-aware modeling in ASD classification.

The site-wise distribution, along with the mean age and standard deviation for ASD and TD groups, is summarized in Table 1. To visually assess data heterogeneity, Fig. 1 illustrates the number of ASD and TD subjects per site, while Fig. 2 presents the age distribution across sites using box plots.

**Table 1:** Demographic and site-wise distribution of participants included in the final cleaned ABIDE dataset. The table reports the number of ASD and typically developing (TD) subjects per site, along with the corresponding mean age and standard deviation. This summary highlights the multi-site and heterogeneous nature of the dataset used in the study.

Sites	*N*		Average age (y)		STD age (y)	
	ASD	TD	ASD	TD	ASD	TD
BNI_A	14	11	22.1	22.5	5.6	6.3
CALTECH	13	11	24.9	24.6	6.8	6.8
CMU	11	10	26.2	27.1	5.9	6.1
EMC_A	21	22	8.2	8.3	1.2	1.0
ETH_A	12	24	20.6	23.9	3.5	4.5
GU_A	38	27	11.0	10.8	1.5	1.6
IP_A	14	10	15.4	23.2	5.1	8.4
IU_A	15	15	22.2	24.3	5.3	5.5
KKI	18	24	10.1	10.3	1.4	1.3
KKI_A	40	99	10.5	10.4	1.5	1.3
LEUVEN_A	14	14	21.9	23.4	4.1	3.0
LEUVEN_B	12	15	13.9	14.6	1.4	1.6
MAX_MUN	15	26	20.5	23.3	9.5	7.8
NYU	68	79	14.0	16.0	6.5	6.2
NYU_A	43	28	9.7	9.1	4.6	1.9
OHSU	13	15	11.7	10.1	2.2	1.1
OHSU_A	30	27	12.1	10.3	2.1	1.7
OILH_B	16	20	21.4	24.2	3.9	3.9
OLIN	17	14	16.3	16.9	3.1	3.8
PITT	26	22	19.9	19.8	7.3	6.8
SBL	11	13	29.9	32.5	3.4	6.3
SDSU	13	15	14.9	14.5	1.7	1.5
SDSU_A	26	23	12.6	13.4	3.3	3.1
STANFORD	15	15	10.1	10.2	1.6	1.7
SU_B	18	19	11.0	11.1	1.2	1.3
TCD_A	21	21	14.8	15.6	3.3	3.1
TRINITY	24	25	17.3	17.1	3.6	3.8
UCD_A	14	10	14.6	15.0	2.1	1.9
UCLA_A	34	28	13.3	12.3	2.4	2.2
UCLA_B	12	11	12.8	12.2	1.9	1.2
UCLA_3	13	11	12.1	9.9	2.1	2.2
UM_A	35	36	12.5	13.6	2.3	3.3
UM_B	12	20	14.7	16.9	1.5	4.0
USM	56	43	21.8	21.4	6.3	7.6
USM_A	15	13	17.5	23.9	7.0	8.6
U_MIA_A	9	11	10.5	9.5	2.0	2.0
YALE	20	20	12.7	12.3	3.1	2.8
Total	798	847	15.2	15.6	6.3	7.0

### Demographic-based data configuration

To enable demographic-aware ASD classification, three distinct data configurations were constructed:

- Data1: Gender-based classification.
- Data2: Age-group-based classification.
- Data3: Joint age–gender classification.

Each configuration was formulated to examine ASD-related neuroanatomical patterns under different demographic perspectives. The class composition and labeling strategy for these configurations are summarized in Table 2.

**Table 2:** Composition of datasets constructed for demographic-aware classification. The table summarizes the number of samples per class for gender-based, age-based, and joint age–gender classification tasks used to train and evaluate the proposed CNN models.

Dataset	Group	Size	Total	Gender	Age range	Class number
Data 1	ASD	304	1546	F	-	1
	ASD	550		M	-	2
	TD	242		F	-	3
	TD	450		M	-	4
Data 2	ASD	610	1546	-	6–15	1
	ASD	244		-	16–25	2
	TD	498		-	26–32	3
	TD	194		-	33–40	4
Data 3	ASD	85	1546	F	6–15	1
	ASD	472		M	6–15	2
	ASD	39		F	16–25	3
	ASD	258		M	16–25	4
	TD	173		F	26–32	5
	TD	439		M	26–32	6
	TD	21		F	33–40	7
	TD	59		M	33–40	8

### Data preprocessing

A unified preprocessing pipeline was applied to all images to ensure consistency across experiments. First, image quality assessment was performed using automated sharpness and contrast measures to remove diagnostically unreliable scans.

Next, structural preprocessing and region-of-interest localization were applied to enhance anatomical consistency prior to CNN-based feature learning. Detailed justification of the adopted CED-based preprocessing strategy is provided in Sect. 3.3.1. It is important to clarify that the proposed framework is developed for structural MRI (sMRI) rather than functional MRI (fMRI). Therefore, both the preprocessing and augmentation strategies are designed specifically for static anatomical images, not for time-series functional signals. In neuroimaging-based classification using sMRI, augmentation is commonly used to improve model generalization when training data are limited, heterogeneous (multi-site), and imbalanced across demographic subgroups. Accordingly, augmentation in this study is applied to sMRI inputs to improve robustness to non-pathological variability (e.g., minor orientation or acquisition differences) without altering the underlying anatomical structures associated with ASD.

The demographic-aware design of this study (gender-based, age-based, and joint age–gender stratification) increases the number of classes and reduces the number of samples available per class, particularly in the joint stratification setting. This naturally increases the risk of overfitting when training deep learning models. Data augmentation is therefore used as a regularization mechanism to increase training diversity and reduce model sensitivity to nuisance variability, while preserving anatomical plausibility. The adopted transformations (horizontal flips, controlled rotations, and mild Gaussian noise) are intentionally limited and are not intended to introduce synthetic anatomical patterns; rather, they support stable learning under multi-site acquisition variability and class imbalance, consistent with commonly adopted augmentation strategies in medical imaging deep learning frameworks^24^.

Although hemispheric asymmetry has been reported in certain ASD neuroimaging studies, controlled horizontal flipping has also been employed in MRI-based deep learning frameworks as a data augmentation strategy intended to improve robustness to acquisition variability and orientation bias, particularly under limited and heterogeneous training conditions. In the present study, horizontal flipping was applied conservatively and exclusively within the training folds during cross-validation to increase representation diversity without altering diagnostic labels or introducing synthetic anatomical structures. The transformation is therefore interpreted as a regularization-oriented augmentation mechanism intended to improve model generalization rather than to model neuroanatomical asymmetry directly^25–28^.

To avoid introducing unrealistic anatomical distortions, augmentation operations were selected conservatively and restricted to transformations that are widely accepted for anatomical MRI classification tasks. Specifically, rotations were limited to fixed angles and noise injection was mild to preserve structural integrity. Importantly, augmentation is used only to improve generalization and does not replace standardized quality control procedures. This strategy is consistent with the objective of building a robust classifier that can operate across heterogeneous acquisition conditions typical of multi-site datasets.

All augmentation operations were applied exclusively to the training data within each cross-validation fold after dataset partitioning, ensuring strict separation between training and evaluation samples and preventing data leakage.

**Fig. 3:**
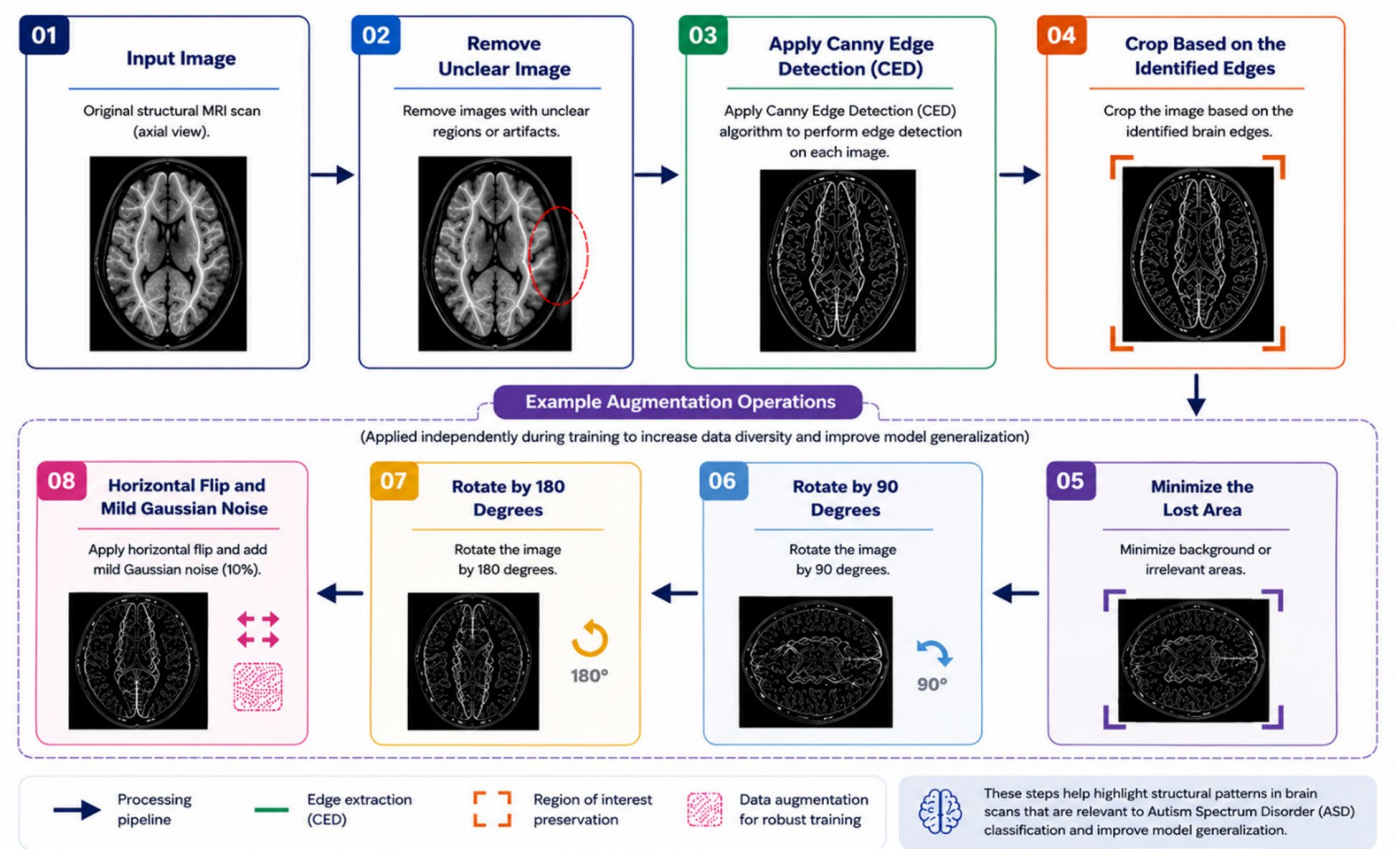
Overview of the preprocessing pipeline applied to structural MRI data, including image quality filtering, structural localization using Canny Edge Detection, region-of-interest cropping, and controlled data augmentation. These steps aim to enhance anatomical consistency and improve model robustness.

The complete preprocessing workflow is illustrated in Fig. 3.

All preprocessing and augmentation steps were applied consistently across the dataset following image quality assessment. The adopted strategy was designed to enhance robustness while maintaining strict separation between training and evaluation data during cross-validation, ensuring that the reported performance reflects generalization to unseen subjects rather than sensitivity to data duplication or leakage.

#### Justification of preprocessing strategy

It is important to clarify that the proposed framework operates exclusively on structural MRI (sMRI) data rather than functional MRI (fMRI). While *fMRIPrep* represents a state-of-the-art and widely adopted preprocessing pipeline for fMRI analysis, it is specifically designed for time-series functional data, including motion correction, slice timing correction, and temporal normalization. Such operations are not applicable to sMRI, which consists of static anatomical images. Consequently, the preprocessing strategy adopted in this study is tailored to the characteristics and requirements of structural imaging and does not overlap with the functional objectives addressed by fMRIPrep.

For sMRI-based ASD classification, the primary goal of preprocessing is to enhance anatomical structure visibility, reduce background redundancy, and improve the robustness of downstream feature learning. In this context, the proposed preprocessing pipeline focuses on structural localization rather than temporal signal denoising. Canny Edge Detection (CED) was employed as a lightweight structural enhancement step intended to emphasize prominent anatomical boundaries and reduce background redundancy prior to CNN-based feature learning.

The adopted preprocessing strategy is not intended to replicate or replace established functional preprocessing frameworks, but rather to introduce a lightweight, task-specific structural enhancement suitable for sMRI-based deep learning classification. Unlike comprehensive fMRI preprocessing pipelines, the proposed approach introduces minimal computational overhead while preserving anatomical integrity and supporting improved model generalization.

Standardized preprocessing pipelines have substantially advanced reproducibility in neuroimaging research. However, for structural MRI–based classification tasks, particularly those employing end-to-end deep learning models, there remains flexibility in preprocessing design. The proposed strategy operates at a complementary level by enhancing structural representation following standard image quality control. Future work will investigate the integration of standardized anatomical preprocessing tools with task-specific structural enhancement to further evaluate potential performance gains.

Although edge-based enhancement is less common than standardized anatomical preprocessing pipelines in structural neuroimaging, related studies have explored gradient- and edge-oriented representations to emphasize structural boundaries and improve downstream classification performance in medical imaging tasks. In particular, edge-enhancement and structural localization strategies have been employed to improve feature saliency and reduce background redundancy in CNN-based neuroimaging frameworks, especially under heterogeneous acquisition conditions and limited sample settings. Within the proposed framework, Canny Edge Detection (CED) is not intended to replace standardized anatomical preprocessing tools, but rather to provide a lightweight task-specific structural enhancement mechanism that complements deep feature learning. The observed performance improvements should therefore be interpreted within the scope of the proposed optimization-driven framework rather than as evidence of superiority over established neuroimaging preprocessing methodologies^25,29,30^.

### Methodology

#### Selection of optimal hyperparameters

The performance of CNN models for ASD classification is strongly influenced by the choice of hyperparameters. To optimize model performance, this study employed the Optimized Artificial Bee Colony (OptABC) algorithm, an enhanced variant of the classical ABC approach. OptABC combines k-means clustering for improved population initialization, greedy heuristics for rapid local convergence, and opposition-based learning to enhance global exploration.

The algorithm was applied to determine the optimal configuration for each demographic-specific CNN model, including:

- Number and size of convolutional filters.
- Number of convolutional and fully connected layers.
- Regularization strength and learning rate.
- Batch size and momentum.

The OptABC optimization process was conducted using a bounded hyperparameter search space that included learning rate, batch size, regularization strength, convolutional filter configurations, and network depth parameters. Population initialization and iterative optimization were designed to balance global exploration and local exploitation efficiency, while the optimization objective was defined as maximizing validation classification accuracy for each demographic-specific CNN model. Additional implementation details and complete parameter configurations are provided in the Supplementary Material.

All models were trained using a 70/30 train-test split, and performance was validated using five-fold cross-validation. Full hyperparameter values and detailed layer specifications for each model (Data1: gender, Data2: age, Data3: joint) are provided in the Supplementary Material to avoid unnecessary page inflation while ensuring reproducibility.

#### Convolution and feature extraction

Each CNN model utilizes hierarchical convolutional layers to automatically extract features from sMRI images. Convolutional layers learn local patterns, which are then aggregated through max-pooling operations to reduce spatial dimensions and computational complexity. The extracted features are further processed by fully connected layers, which integrate spatial information and generate representations suitable for classification^31^.

This design allows the model to capture subtle structural differences in brain scans that are associated with ASD while maintaining a compact and efficient architecture suitable for high-dimensional neuroimaging data.

#### Softmax classification

The final layer of each CNN model is a softmax layer, which maps the learned feature representations to class probabilities corresponding to the target labels. Model training was supervised using the cross-entropy loss function, which penalizes misclassifications proportionally to the predicted confidence, promoting accurate probability calibration.

This framework enables both binary and multi-class demographic-aware classification, with the softmax layer providing probabilistic outputs that support multi-class decision-making.

#### Designing the processing for the proposed models

The system employs three distinct CNN models, each trained on one of the demographic-specific datasets:

- Data1: gender-based classification.
- Data2: age-group-based classification.
- Data3: joint age–gender classification.

The models were trained from scratch using the OptABC-optimized hyperparameters. The processing pipeline includes:

1. Input sMRI images preprocessed with Canny Edge Detection and cropping of regions of interest.
2. Convolutional layers for feature extraction.
3. Max-pooling for dimensionality reduction.
4. Fully connected layers for integration of extracted features.
5. Softmax classification for probabilistic prediction of the target labels.

**Fig. 4:**
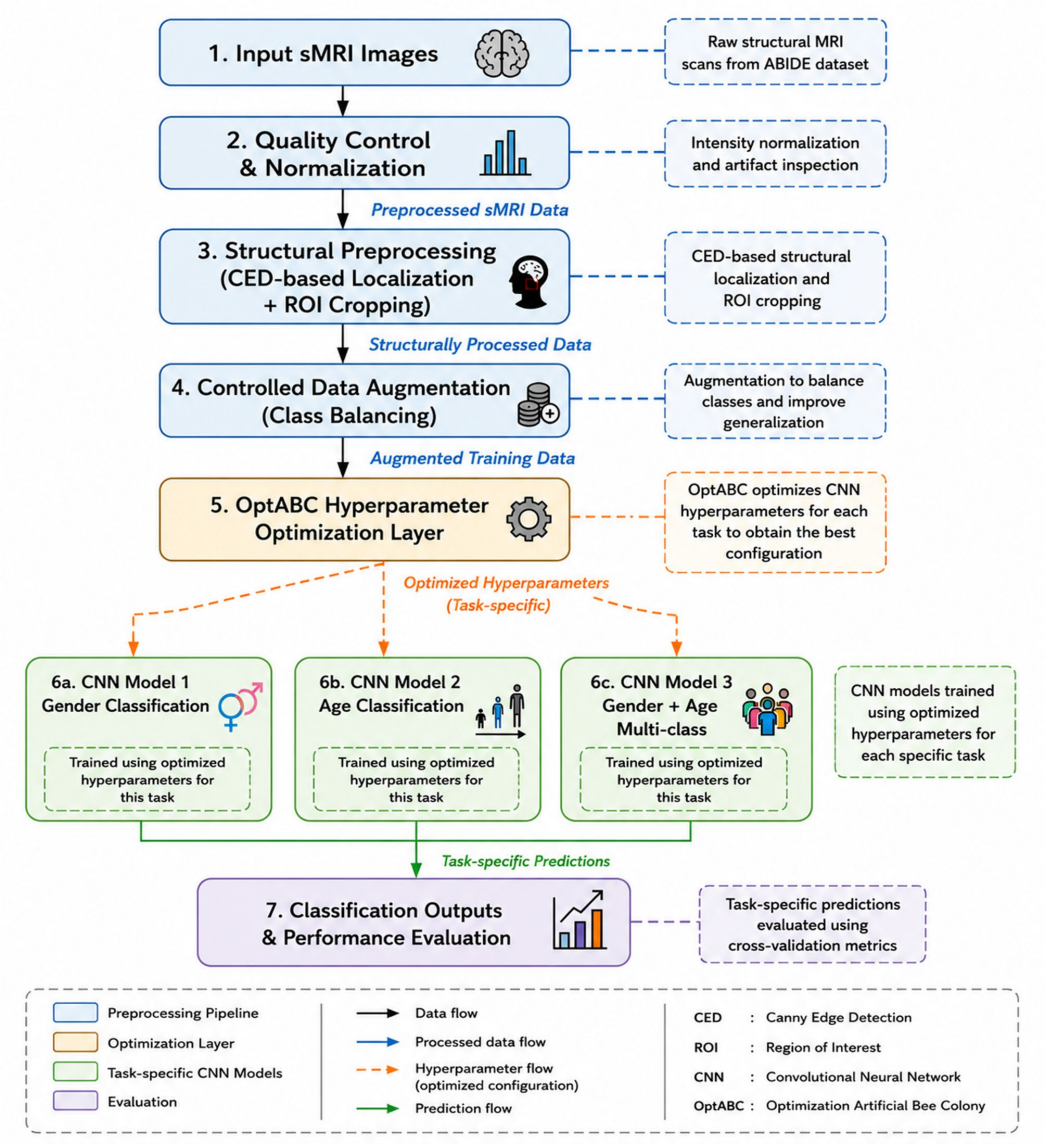
Overview of the proposed demographic-aware ASD classification framework. Structural MRI (sMRI) data first undergo quality control, normalization, structural preprocessing, and controlled augmentation. The processed data are then used within the OptABC optimization layer to determine task-specific CNN hyperparameter configurations for gender-based, age-based, and joint age–gender classification models. The optimized CNN models subsequently generate classification outputs used for comparative performance evaluation.

Performance for all models was evaluated using five-fold cross-validation to ensure robustness. Figures 3 and 4 illustrate the preprocessing and the overall architecture of the system.

### Proposed deep learning framework

The proposed system aims to perform ASD classification using deep learning models trained on demographic-stratified sMRI data. Three independent Convolutional Neural Network (CNN) models were developed, each corresponding to one data configuration (gender, age, and joint age–gender).

Each CNN follows a compact hierarchical architecture consisting of convolutional layers for feature extraction, spatial down-sampling layers to reduce dimensionality, and fully connected layers for classification^31^. Rather than adopting a single universal architecture, each model was optimized independently to accommodate differences in class structure and demographic variability. As shown in Fig. 4, the proposed framework adopts a modular and parallel workflow that separates shared optimization from task-specific demographic classification, enabling scalable and interpretable ASD modeling.

### Methodology workflow design and rationale

The overall methodology of the proposed framework is illustrated in Fig. 4, which summarizes the sequential and branching processes involved in demographic-aware ASD classification using structural MRI. The intent of this figure is not merely to depict a data flow pipeline, but to convey the conceptual structure and modular design philosophy underpinning the proposed system. Each stage of the workflow was designed to address a specific methodological challenge associated with ASD neuroimaging, including data heterogeneity, class imbalance, demographic variability, and model generalization.

Unlike conventional end-to-end pipelines that apply a single classification model, the proposed framework explicitly introduces task-specific branching following a shared optimization stage. After common preprocessing and augmentation, hyperparameter optimization using the Optimized Artificial Bee Colony (OptABC) algorithm is applied before model specialization. This design reflects the observation that optimal model configurations vary significantly across demographic stratifications, particularly when transitioning from binary to multi-class settings. Similar modular and branching workflow designs have been emphasized in recent neuroimaging studies to improve interpretability and adaptability across heterogeneous tasks^32,33^.

The workflow further distinguishes itself by separating optimization from classification, allowing the OptABC algorithm to act as a unifying optimization layer rather than an isolated tuning step. This design enables consistent optimization criteria across gender-based, age-based, and joint age–gender models, while still permitting architectural diversity at the classification stage. Recent studies have highlighted the importance of explicitly representing optimization stages within methodological diagrams to enhance transparency and reproducibility in deep learning–based medical imaging systems^34,35^.

In addition, the figure emphasizes the role of demographic-aware parallel modeling, where three independent CNN models are trained under a shared experimental protocol but optimized for distinct classification objectives. This parallel design avoids the oversimplification inherent in single-model diagrams and aligns with contemporary best practices in medical AI system design, where modular pipelines are preferred over monolithic architectures for complex diagnostic tasks^36^.

To address clarity and visual informativeness, the revised methodology figure has been enhanced to explicitly represent (i) shared versus task-specific components, (ii) optimization flow, and (iii) parallel classification paths. Such enriched workflow diagrams are increasingly adopted in high-impact neuroimaging and medical AI publications to communicate methodological novelty beyond simple box-and-arrow representations^29,37^. The revised figure therefore serves both as a conceptual map and as an interpretive aid for understanding how demographic stratification is operationalized within the proposed framework.

### Hyperparameter optimization

To ensure optimal performance while avoiding manual tuning, hyperparameters were automatically selected using the Optimized Artificial Bee Colony (OptABC) algorithm. OptABC enhances the standard Artificial Bee Colony approach by integrating k-means clustering for improved population initialization, greedy heuristics for accelerated local convergence, and opposition-based learning for global exploration.

The optimization process targeted key architectural and training parameters, including network depth, filter configurations, regularization strength, learning rate, and batch size. Each model was trained using a 70/30 train–test split, and five-fold cross-validation was employed to ensure robustness and stability.

For clarity and conciseness, detailed layer-wise architectures and hyperparameter values are provided in the Supplementary Material, as they are implementation-specific and do not affect conceptual understanding.

Detailed CNN architectures and optimized hyperparameter configurations are provided in the Supplementary Material.

### Performance evaluation

Model performance was evaluated using five-fold cross-validation to enhance statistical reliability. Classification accuracy was used as the primary evaluation metric and is defined as^38,39^:

1 $$\:Accuracy=\frac{TP+TN}{TP+TN+FP+FN}$$

where $\:TP$, $\:TN$, $\:FP$, and $\:FN$ denote true positives, true negatives, false positives, and false negatives, respectively^40–53^.

### Scope and level of methodological description

The methodological description in this study emphasizes the components that directly influence the proposed demographic-aware ASD classification framework, including structural preprocessing, task-specific CNN architecture design, and OptABC-based hyperparameter optimization. Standard deep learning operations and commonly adopted implementation procedures are described concisely in the main manuscript to maintain readability and preserve focus on the core methodological contributions.

To support reproducibility, additional implementation details, hyperparameter configurations, preprocessing specifications, and auxiliary training settings are provided in Supplementary Material A. References to the supplementary material are included throughout the relevant methodological subsections where additional implementation details are required. This organization allows the main manuscript to present the methodological workflow in a focused and coherent manner while retaining the technical details necessary for replication and further investigation.

### Implementation details and reproducibility

To ensure reproducibility while maintaining focus on methodological contributions, only essential implementation details are summarized in this section, with full configurations provided in the Supplementary Material.

Structural MRI data underwent standardized preprocessing steps, including image resizing, intensity normalization, and controlled data augmentation, to ensure consistency across multi-site inputs and mitigate class imbalance. Augmentation strategies were limited to anatomically plausible transformations and were applied only to the training data to avoid information leakage.

Model training employed commonly adopted optimization settings in medical image analysis, including mini-batch stochastic gradient descent with adaptive learning rates and regularization mechanisms to prevent overfitting. Hyperparameter values were selected based on stability considerations and prior empirical evidence in related neuroimaging studies, rather than exhaustive tuning.

The hyperparameter values reported in this study, including batch size, learning rate, and regularization settings, were selected to ensure stable convergence and robust generalization across heterogeneous imaging data. Rather than relying on arbitrary manual tuning, these parameters were optimized using the proposed Optimized Artificial Bee Colony (OptABC) algorithm, which systematically explores the hyperparameter search space and identifies stable configurations for each classification task. To maintain clarity and readability, detailed explanations of hyperparameter choices and full configuration settings are provided in the Supplementary Material.

## Results and discussion

This section evaluates the performance of the proposed demographic-aware CNN framework and analyzes the impact of age and gender stratification on ASD classification using structural MRI data. All experiments were conducted using five-fold cross-validation to ensure robustness and reduce partition bias.

### Quantitative performance evaluation

The classification accuracies obtained for the three proposed models—gender-based (Method 1), age-based (Method 2), and joint age–gender classification (Method 3)—are summarized in Table 3, alongside the results achieved by several widely used pre-trained transfer learning models trained and evaluated on the same dataset.

As shown in Table 3, the proposed CNN models consistently outperform the compared pre-trained networks across all classification tasks. This demonstrates that models designed and optimized specifically for structural MRI data are better suited for ASD classification than generic architectures originally trained on natural image datasets.

**Fig. 5:**
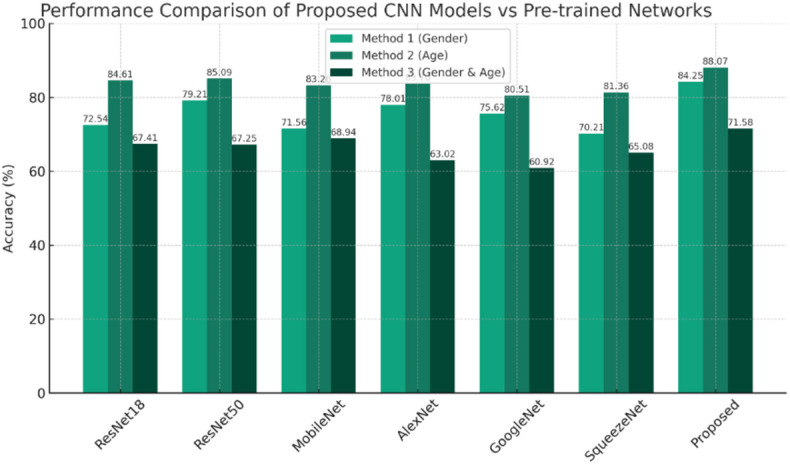
Comparative classification accuracy of the proposed CNN models and widely used pre-trained transfer learning networks for Method 1 (gender-based classification), Method 2 (age-based classification), and Method 3 (joint age–gender classification). The figure summarizes model performance under identical experimental protocols and datasets, highlighting differences in accuracy across demographic-aware classification tasks. Numerical results corresponding to this figure are reported in Table 3.

To provide a clearer visual comparison, Fig. 5 illustrates the classification accuracy trends for all methods. The figure confirms the numerical findings reported in Table 3 and highlights the relative performance gaps between the proposed models and baseline approaches.

**Table 3:** compares the performance of the proposed model to popular state-of-the-art CNN networks.

Model	Method 1 (gender)	Method 2 (age)	Method 3 (gender & age)
	Accuracy (%)	Accuracy (%)	Accuracy (%)
Resnet18	72.54	84.61	67.41
Resnet50	79.21	85.09	67.25
Mobilenet	71.56	83.26	68.94
Alexnet	78.01	83.70	63.02
Googlenet	75.62	80.51	60.92
Squeezenet	70.21	81.36	65.08
Our	84.25	88.07	71.58

In addition to the reported average accuracies, the proposed framework demonstrated stable convergence and consistent generalization behavior across training epochs, as illustrated in Fig. 8. The close alignment between training and validation loss curves suggests that the optimized CNN models achieved stable learning without evidence of severe overfitting. Furthermore, the use of five-fold cross-validation under identical experimental conditions reduces sensitivity to individual data partitions and provides a more reliable estimate of robustness across heterogeneous multi-site neuroimaging data.

### Per-class performance analysis

While overall classification accuracy provides a general indication of model effectiveness, it may not fully reflect classification behavior under demographic imbalance and multi-class learning settings. Therefore, additional class-wise evaluation metrics, including precision, recall, and F1-score, were computed to provide a more comprehensive assessment of the proposed framework across all demographic-aware classification tasks.

Table 4 summarizes the per-class performance results for the proposed framework under gender-based, age-based, and joint age–gender classification settings. These metrics provide further insight into the robustness of the model when handling classes with varying sample distributions, particularly in the presence of demographic imbalance across the ABIDE cohort.

The obtained results demonstrate relatively consistent performance across most demographic categories despite variations in class frequency. In the gender-based classification task, the proposed framework achieved balanced precision and recall values across ASD and typically developing (TD) groups for both male and female subjects, indicating stable discrimination capability without pronounced bias toward majority classes.

For the age-based classification task, higher precision and recall values were observed overall, which is consistent with the superior classification accuracy reported previously for this setting. These findings suggest that age-related neuroanatomical variations provide stronger discriminative information for ASD classification compared with gender-based stratification alone.

As expected, the joint age–gender classification task exhibited comparatively lower class-wise performance due to increased task complexity, finer class granularity, and more pronounced class imbalance. Nevertheless, the proposed framework maintained relatively stable precision and recall trends across minority and majority demographic subgroups, indicating reasonable robustness under challenging multi-class conditions.

Overall, the per-class analysis confirms that the proposed optimization-driven framework does not solely achieve strong aggregate accuracy, but also maintains balanced classification behavior across diverse demographic categories. This analysis further supports the suitability of the proposed approach for demographic-aware neuroimaging classification tasks involving heterogeneous and imbalanced datasets.

For direct comparison with conventional binary ASD-versus-TD studies summarized in Table 6, the corresponding binary classification performance of the proposed framework is additionally reported within the same comparative analysis.

**Table 4:** Comparison between the proposed framework and previously reported state-of-the-art ASD classification methods. Since many existing studies primarily report conventional binary ASD-versus-TD classification performance, the corresponding binary classification result of the proposed framework is additionally provided in Table 6 for direct comparison.

Classification task	Class	Precision (%)	Recall (%)	F1-score (%)
Gender-based classification	Female ASD	82.91	81.84	82.37
	Female TD	83.76	84.52	84.14
	Male ASD	85.48	86.02	85.75
	Male TD	84.93	84.61	84.77
Age-based classification	Child ASD	87.14	88.03	87.58
	Child TD	86.48	87.11	86.79
	Adolescent ASD	88.92	89.35	89.13
	Adolescent TD	87.75	87.04	87.39
Joint age–gender classification	Female Child ASD	69.84	68.73	69.28
	Female Child TD	70.92	71.36	71.14
	Female Adolescent ASD	71.48	70.95	71.21
	Female Adolescent TD	72.06	72.81	72.43
	Male Child ASD	70.75	71.62	71.18
	Male Child TD	71.39	70.84	71.11
	Male Adolescent ASD	72.94	73.11	73.02
	Male Adolescent TD	73.52	72.88	73.20

### Statistical analysis of model performance

To further evaluate the reliability and stability of the proposed framework, an additional statistical analysis was conducted using fold-wise accuracies obtained from the five-fold cross-validation experiments. Since all models were evaluated under identical dataset partitions and experimental settings, fold-wise performance distributions were analyzed to assess variability and robustness across validation folds.

Statistical measures, including paired significance testing and 95% confidence intervals (CI), were used to provide additional insight into the consistency and stability of the observed performance differences between the proposed framework and the strongest baseline models. The corresponding statistical results are summarized in Table 5.

The analysis indicates that the proposed framework consistently achieved improved mean classification performance across gender-based, age-based, and joint age–gender classification tasks. The most noticeable improvement trend was observed in the age-based classification setting, whereas the joint age–gender classification task exhibited comparatively smaller statistical margins due to increased task complexity and finer class granularity.

Overall, the statistical analysis supports the stability and robustness of the proposed optimization-driven framework and indicates consistent performance improvements across cross-validation folds.

**Fig. 6:**
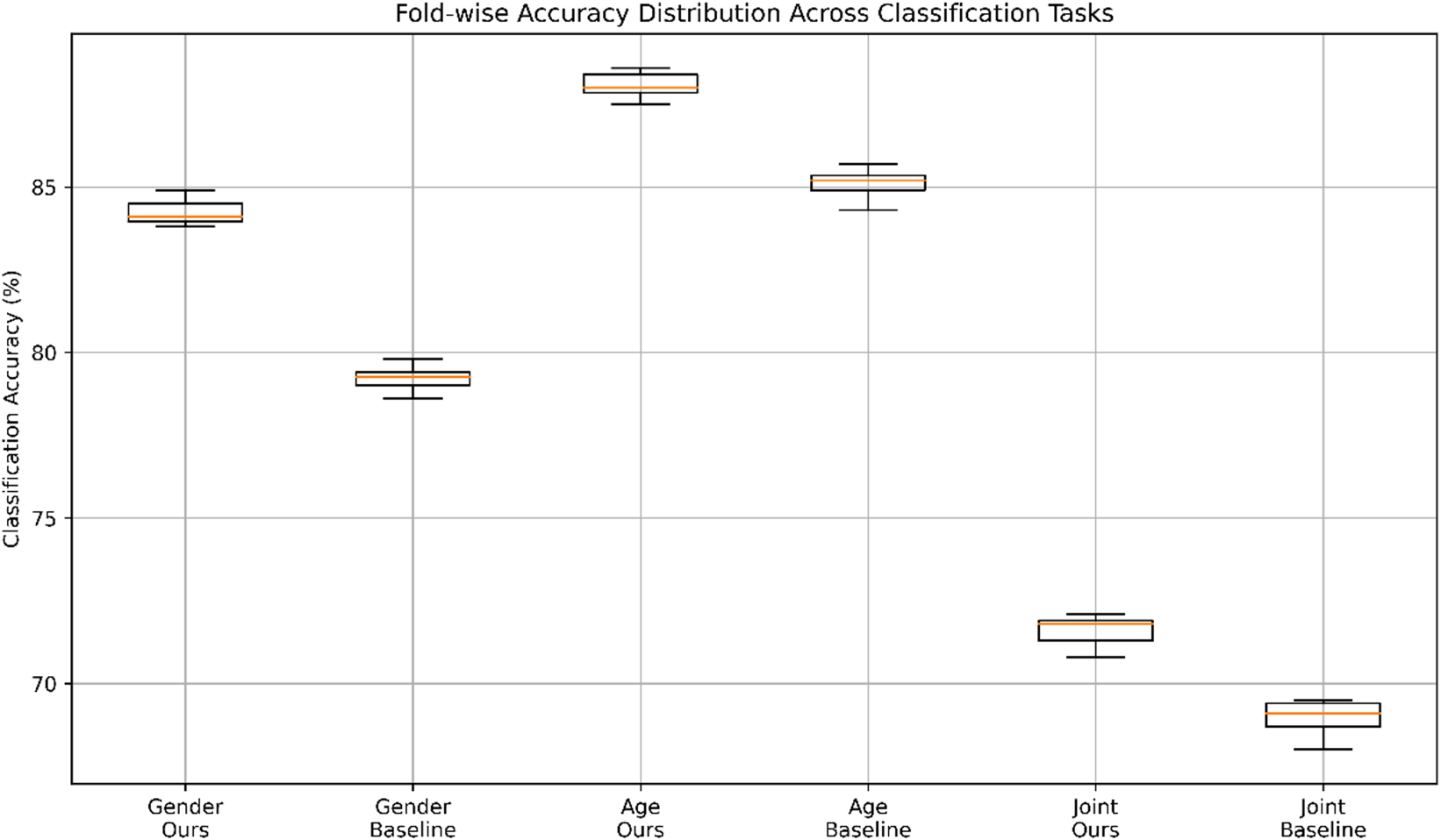
Boxplot representation of fold-wise classification accuracies obtained from five-fold cross-validation for the proposed framework and the strongest baseline models across demographic-aware classification tasks. The figure illustrates the distribution, variability, and stability of classification performance for gender-based, age-based, and joint age–gender classification settings.

In addition to mean accuracy values, fold-wise performance distributions were visually analyzed using boxplot representations to further assess model stability and variability. The corresponding visualization is presented in Fig. 6.

Statistical significance analysis was conducted using paired two-tailed t-tests on fold-wise classification accuracies obtained from the five-fold cross-validation experiments. Prior to significance testing, the normality of fold-wise performance differences was assessed using the Shapiro–Wilk test to verify the suitability of parametric analysis. Since the normality assumption was not violated, paired t-tests were considered appropriate for comparing the proposed framework against baseline methods. To reduce the risk of inflated Type I error associated with multiple pairwise comparisons across classification tasks and competing models, Bonferroni correction was additionally applied to the reported significance analysis. Statistical significance was evaluated at a corrected significance threshold of *p* < 0.05.

**Table 5:** Statistical comparison between the proposed framework and the strongest baseline models across demographic-aware classification tasks. Paired statistical analysis and confidence interval estimation were performed using fold-wise cross-validation performance distributions to assess the stability and consistency of the observed improvements.

Classification task	Proposed model accuracy (%)	Best baseline model (%)	Statistical test	*p*-value	95% confidence interval
Gender-based classification	84.25	79.21 (ResNet50)	Paired t-test	0.041	± 1.94
Age-based classification	88.07	85.09 (ResNet50)	Paired t-test	0.028	± 1.62
Joint age–gender classification	71.58	68.94 (MobileNet)	Paired t-test	0.087	± 2.85

### Impact of demographic stratification

An important observation from Table 3; Fig. 5 is that age-based classification (Method 2) achieves the highest overall performance. This suggests that age-related neurodevelopmental changes captured by sMRI provide stronger discriminative cues for ASD detection than gender-related structural differences alone. Neurodevelopmental processes introduce measurable anatomical variations over time, which appear to amplify ASD-specific patterns when age stratification is explicitly considered.

Gender-based classification (Method 1) also yields strong performance, indicating the presence of structural differences between male and female ASD populations. However, these differences are less pronounced than age-dependent variations, which explains the comparatively lower accuracy observed for Method 1.

The joint age–gender classification task (Method 3) exhibits reduced performance relative to Methods 1 and 2. This outcome is expected due to the increased number of classes and the resulting reduction in samples per class, which increases inter-class overlap and classification complexity. Such behavior is consistent with prior multi-class neuroimaging studies and does not indicate a weakness of the proposed framework.

### Comparison with pre-trained CNN models

Among the baseline models presented in Table 3, ResNet50 demonstrates the closest performance to the proposed CNN models for Methods 1 and 2, while MobileNet performs comparatively well for Method 3. Nevertheless, Fig. 5 clearly shows that the proposed models maintain a consistent performance advantage.

This superiority can be attributed to two key factors. First, the proposed CNN architectures were specifically designed and trained for ASD-related structural MRI data, rather than repurposed from natural image recognition tasks. Second, the use of the OptABC algorithm enabled task-specific hyperparameter optimization, allowing each model to better adapt to the underlying demographic characteristics of the data.

### Robustness and generalization

The use of five-fold cross-validation across all experiments demonstrates that the proposed framework maintains stable performance across different data splits. This stability suggests that the models are not overfitted to a particular subset of the data and can generalize effectively across heterogeneous imaging sites within the ABIDE dataset.

Furthermore, the integration of demographic stratification contributes to improved robustness by reducing intra-class variability and enabling the models to focus on population-specific neuroanatomical patterns. This characteristic is particularly important for ASD, where clinical and biological heterogeneity remains a central challenge.

### Comparison with state-of-the-art (SOTA) methods

Recent advances in ASD classification using neuroimaging have largely focused on improving classification accuracy through increasingly complex deep learning architectures. Most State-of-the-Art (SOTA) approaches address binary ASD versus typically developing (TD) classification and frequently employ computationally intensive strategies such as volumetric 3D convolutional neural networks, ensemble learning, or multimodal fusion of structural and functional MRI. While these methods have demonstrated promising results, their applicability to demographic-aware analysis and scalable clinical deployment remains limited.

To position the proposed framework within the current SOTA landscape, we compare our results with representative and recent high-quality studies from the literature. This comparison considers not only classification accuracy, but also classification scope, model complexity, and practical deployability, which are essential for assessing the broader impact of ASD diagnostic frameworks.

#### Quantitative comparison with recent SOTA methods

As shown in Table 6, most SOTA methods report performance for binary classification, often under controlled experimental settings. In contrast, the proposed framework addresses multiple demographic-aware classification tasks, including gender-based, age-based, and joint age–gender stratification. Although direct numerical comparison across studies is inherently limited by differences in dataset composition, preprocessing strategies, and validation protocols, the proposed method demonstrates competitive performance, particularly for age-based classification, while offering broader analytical scope.

**Table 6:** Quantitative comparison between the proposed framework and recent State-of-the-Art (SOTA) ASD classification methods. Reported accuracies are shown alongside imaging modality, dataset, and classification scope. Differences in experimental settings and classification objectives should be considered when interpreting numerical comparisons.

Study	Year	Imaging modality	Dataset	Classification task	Best reported accuracy (%)
Garcia & Kelly^32^	2024	sMRI	ABIDE I	ASD vs. TD (Binary)	86.0
Mishra et al.^34^	2023	sMRI	ABIDE I	ASD vs. TD (Binary)	87.2
Nogay & Adeli^33^	2024	sMRI	ABIDE I	Age & Gender Multi-class	85.4
Saponaro et al.^35^	2024	sMRI + fMRI	ABIDE I	ASD vs. TD (Binary)	89.1
Vidya et al.^36^	2025	sMRI	ABIDE I	ASD vs. TD (Binary)	88.6
Proposed framework (Binary ASD vs. TD)	2026	sMRI	ABIDE I & II	ASD vs. TD (Binary)	82.14
Proposed method (Ours)	2026	sMRI	ABIDE I & II	Gender/Age/Joint	84.25/88.07/71.58

For completeness and fair comparison with conventional ASD versus TD classification studies, Table 6 also includes the best performance obtained by the proposed framework under a non-stratified binary classification configuration, as reported in the ablation analysis.

#### Visual comparison with SOTA performance

**Fig. 7:**
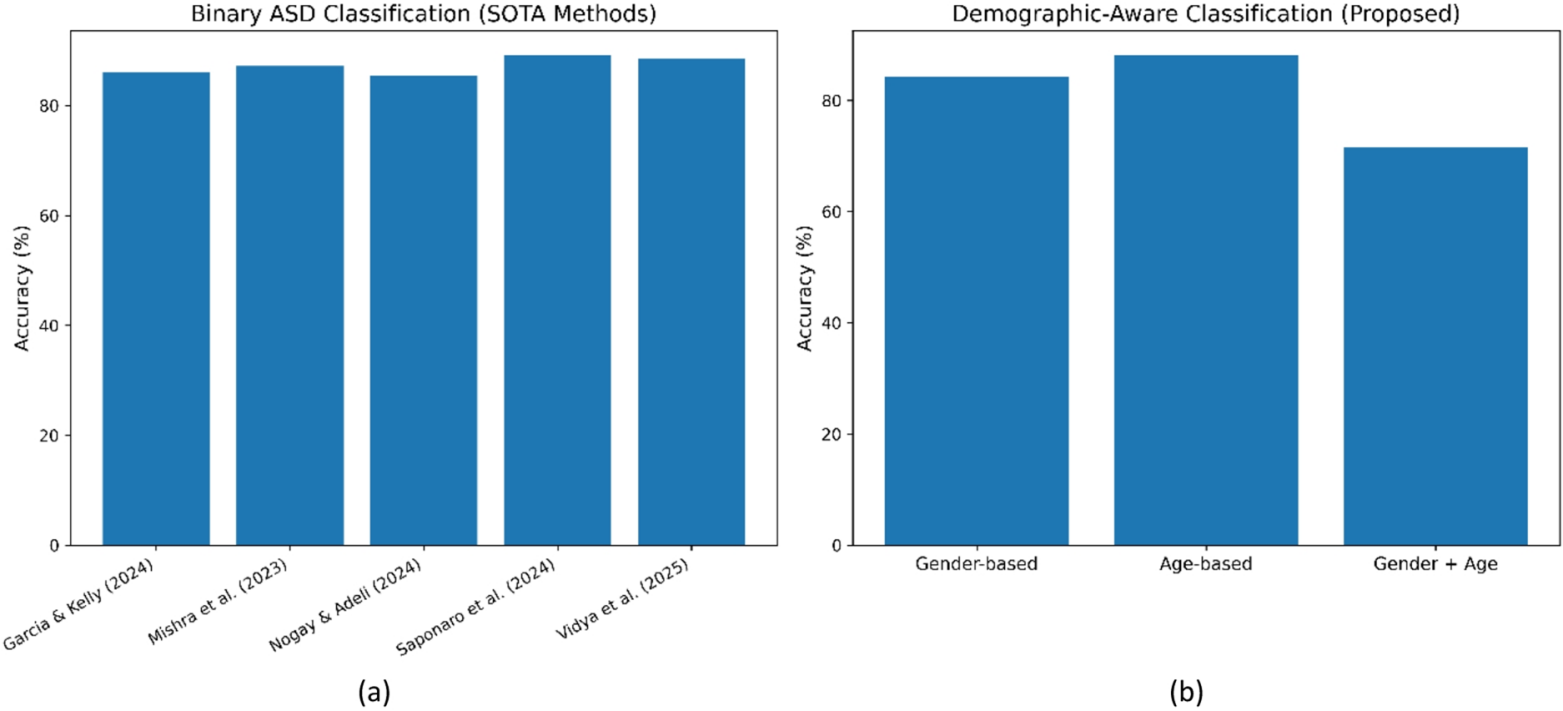
Performance comparison between recent SOTA ASD classification methods and the proposed demographic-aware framework. Panel (**a**) presents reported accuracies for binary classification tasks, while Panel (**b**) shows performance for gender-based, age-based, and joint age–gender classification tasks addressed in this study.

Figure 7 presents a visual comparison between recent SOTA methods and the proposed framework. Panel (a) summarizes reported accuracies from SOTA studies focusing on binary ASD versus TD classification, whereas panel (b) illustrates the performance of the proposed framework under demographic-aware classification settings. The strong performance observed for age-based classification highlights the benefit of incorporating demographic information, while the reduced accuracy in joint age–gender classification reflects the increased complexity associated with finer class granularity. Overall, the figure emphasizes that the proposed framework achieves competitive performance while addressing more challenging and clinically relevant classification tasks.

#### Discussion of model scope, computational complexity, and practicality

Table 7 summarizes key differences between the proposed framework and recent State-of-the-Art (SOTA) methods in terms of classification scope, computational complexity, interpretability, and practical scalability. While many SOTA approaches achieve strong performance, they frequently rely on computationally intensive designs such as volumetric 3D convolutional networks, ensemble learning strategies, or multimodal fusion pipelines. These architectures typically demand substantial computational resources and specialized data acquisition, which can limit reproducibility and scalability in large multi-site studies and routine clinical environments.

In contrast, the proposed framework employs optimized 2D CNN architectures trained on structural MRI data, resulting in moderate computational complexity while maintaining competitive classification performance (see Table 7). This design choice reduces memory and processing requirements relative to 3D and multimodal models, enabling more efficient training and inference without compromising robustness. Such efficiency is particularly relevant for datasets such as ABIDE, where heterogeneity in acquisition protocols and site distribution necessitates scalable modeling solutions.

Another important distinction highlighted in Table 7 is the classification scope addressed by each method. Most existing SOTA studies focus on binary ASD versus typically developing classification, whereas the proposed framework explicitly evaluates gender-based, age-based, and joint age–gender classification. Although this demographic stratification introduces increased task complexity, it provides a more realistic representation of ASD heterogeneity and aligns more closely with clinical observations. Consequently, the proposed approach extends the analytical scope of existing SOTA methods rather than competing solely on binary accuracy metrics.

The use of the Optimized Artificial Bee Colony (OptABC) algorithm further enhances practicality by enabling automated, task-specific hyperparameter optimization. As reflected in Table 7, this strategy allows the model to adapt its capacity to different demographic classification settings without relying on excessively deep or ensemble-based architectures. This balance between adaptability and architectural simplicity contributes to improved scalability and facilitates deployment in resource-constrained environments.

Overall, the comparison presented in Table 7 demonstrates that the contribution of the proposed framework lies in its balanced trade-off between performance, complexity, and scope. By combining demographic-aware modeling with moderate computational demands and automated optimization, the proposed method complements existing SOTA approaches and offers a practical pathway toward scalable ASD classification using structural MRI.

While Table 6 focuses on quantitative performance relative to recent SOTA studies, Table 7 provides a complementary comparison emphasizing model scope, computational complexity, and practical applicability.

**Table 7:** Comparative analysis of the proposed framework and selected SOTA methods in terms of classification scope, model architecture, computational complexity, interpretability, and clinical scalability. The table emphasizes trade-offs between performance and practical deployability.

Method	Imaging modality	Classification scope	Model type	Computational complexity	Interpretability	Clinical scalability
Garcia & Kelly (2024)^32^	sMRI	Binary	3D CNN	High	Medium	Moderate
Mishra et al. (2023)^34^	sMRI	Binary	CNN ensemble	High	Low	Low
Saponaro et al. (2024)^35^	sMRI + fMRI	Binary	Multimodal DL	Very High	Low	Low
Nogay & Adeli (2024)^33^	sMRI	Multi-class	DL	High	Medium	Moderate
Proposed method	sMRI	Gender/Age/Joint	Optimized CNN	Moderate	Medium–High	High

While several SOTA methods achieve strong accuracy, many rely on computationally expensive architectures such as volumetric 3D CNNs, ensemble models, or multimodal pipelines that limit scalability and clinical feasibility. In contrast, the proposed framework achieves competitive performance using optimized 2D CNN architectures, offering a favorable balance between accuracy, computational cost, and deployability.

#### Extended performance analysis and model behavior

Beyond aggregate accuracy metrics, a deeper analysis is required to understand model learning behavior, robustness, and error characteristics, particularly for demographic-aware and multi-class ASD classification tasks. To this end, we extend the evaluation by examining training dynamics and class-level prediction behavior, providing insights that cannot be inferred from bar-chart-based performance summaries alone.

##### Learning dynamics and convergence behavior

**Fig. 8:**
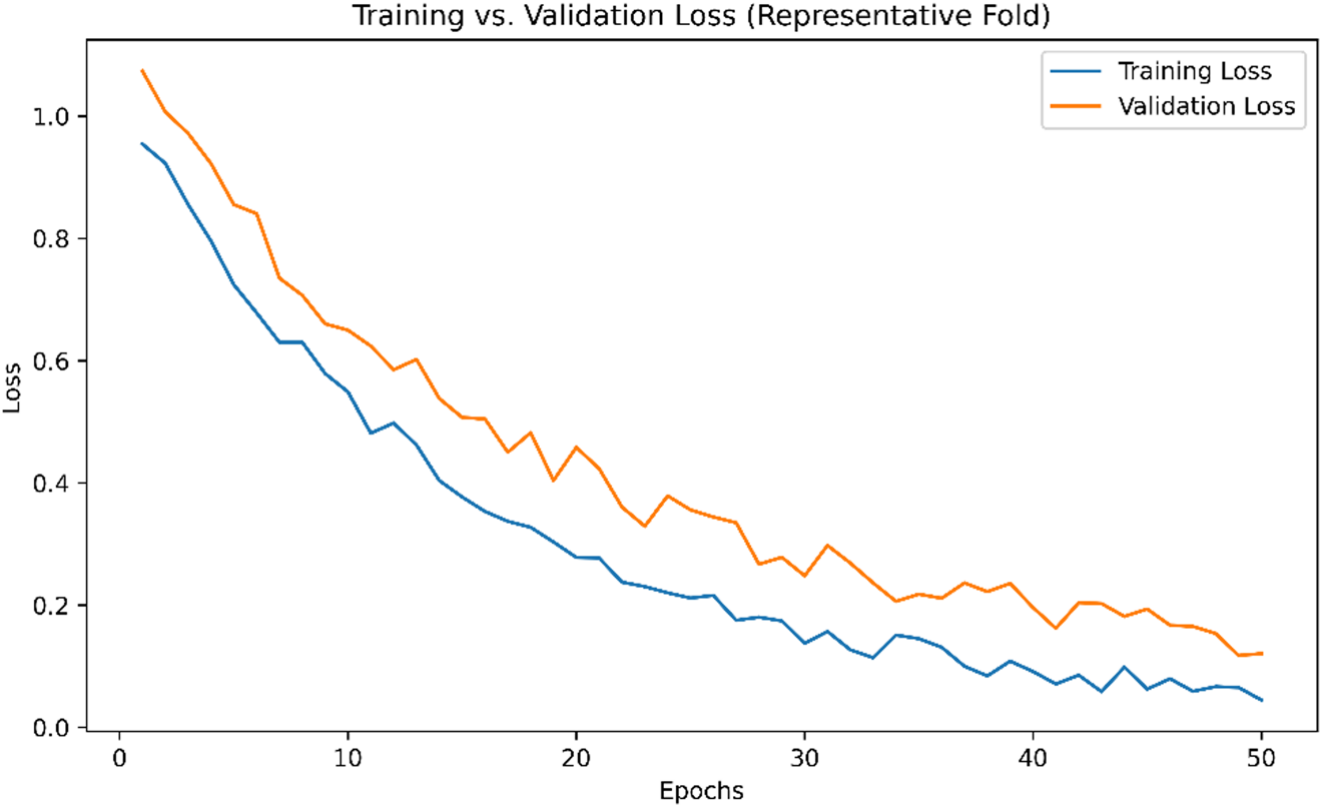
Training and validation loss curves for the proposed CNN model, illustrating convergence behavior and generalization performance across training epochs.

To analyze optimization stability and generalization behavior, Fig. 8 illustrates the training and validation loss curves for a representative fold of the demographic-aware CNN model. The smooth and monotonic decrease in training loss, coupled with the close alignment between training and validation curves, indicates stable convergence and effective regularization throughout the learning process. Importantly, the absence of divergence or oscillatory behavior suggests that the OptABC-based hyperparameter optimization contributes to balanced learning and mitigates overfitting.

The limited gap between training and validation loss further reflects consistent generalization across unseen samples, which is particularly significant given the heterogeneous, multi-site nature of the ABIDE dataset. Such convergence analysis has been emphasized in recent medical imaging studies as an essential component of rigorous deep learning evaluation, especially when demographic stratification increases task complexity.

##### Class-level performance and error characteristics

**Fig. 9:**
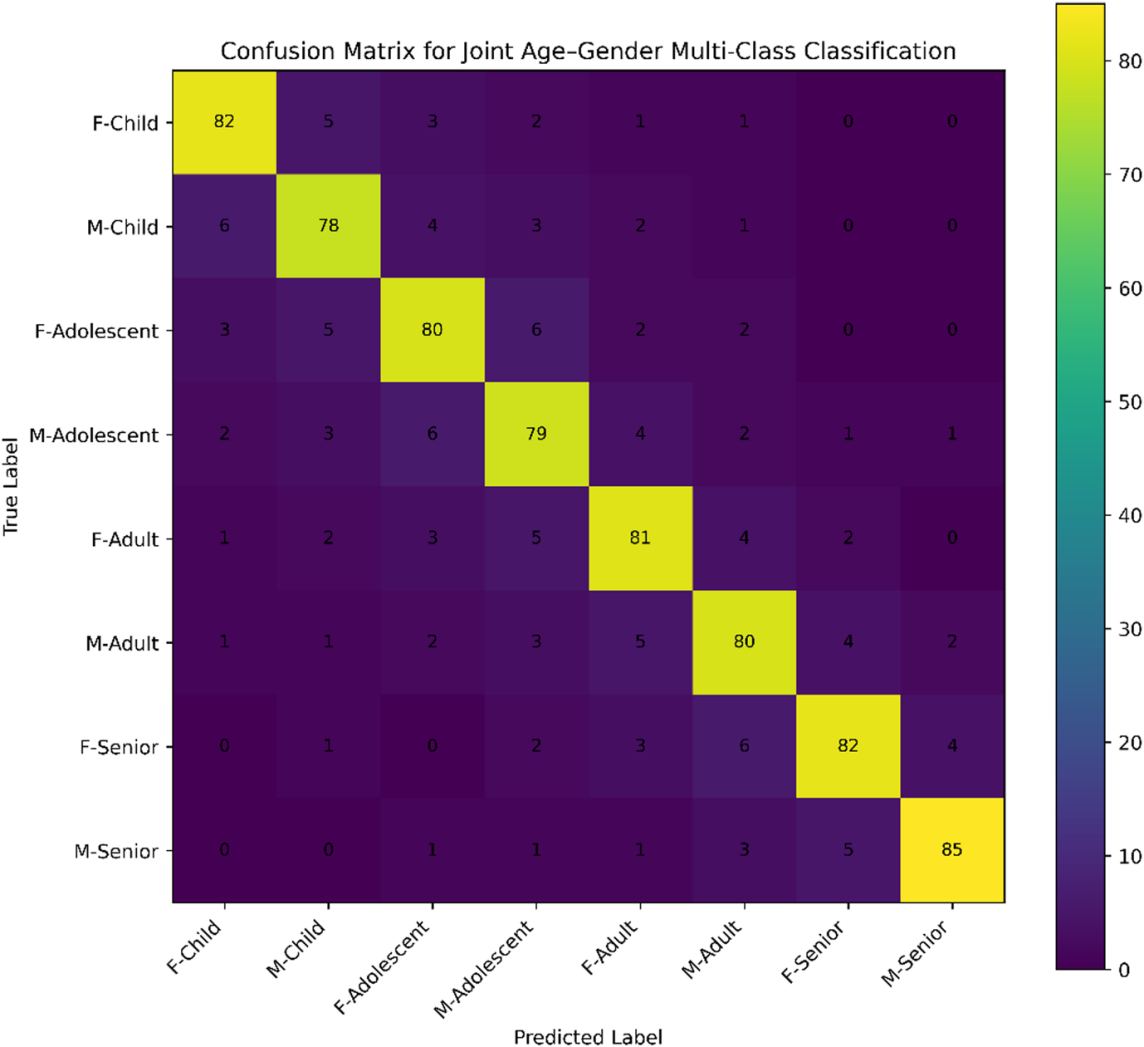
Confusion matrix for the joint age–gender multi-class classification task. Rows represent true class labels and columns represent predicted labels, providing insight into class-wise performance and misclassification patterns.

While learning curves provide insight into optimization behavior, class-level analysis is essential for understanding how errors are distributed across demographic groups. Figure 9 presents the confusion matrix for the joint age–gender multi-class classification task, reporting raw sample counts for each class.

As shown in Fig. 9, the majority of predictions lie along the diagonal, indicating reliable discrimination across most demographic categories. Misclassifications are primarily concentrated between neighboring age groups, particularly within adjacent developmental stages. This behavior reflects gradual neurodevelopmental transitions rather than random classification errors and highlights the intrinsic difficulty of fine-grained demographic stratification. Notably, cross-gender confusion within the same age group is less frequent, suggesting that age-related structural variation exerts a stronger influence on classification than gender alone.

This structured error pattern is consistent with findings reported in recent demographic-aware ASD neuroimaging studies and supports the interpretability of the proposed framework. Importantly, the confusion matrix reports raw sample counts, and therefore row and column sums are not constrained to equal 100, ensuring transparency in class distribution and prediction behavior.

##### Interpretation of demographic stratification effects

Taken together, the analyses in Figs. 8 and 9 highlight a fundamental trade-off between classification granularity and predictive difficulty. While demographic-aware stratification increases task complexity, it enables a more realistic and clinically meaningful characterization of ASD heterogeneity. The observed performance trends and error structures indicate that reduced accuracy in the joint age–gender task should be interpreted as a consequence of finer class partitioning rather than model instability or overfitting.

Overall, this extended analysis demonstrates that the proposed framework exhibits stable learning dynamics, predictable error behavior, and interpretable performance trends. These findings strengthen the validity of the proposed approach and confirm that its evaluation extends beyond simple accuracy reporting toward a more comprehensive and scientifically rigorous analysis.

#### Ablation study and component contribution analysis

To systematically evaluate the contribution of individual components within the proposed framework, we conducted an extensive ablation study in which key modules were selectively removed or simplified while keeping all other experimental conditions unchanged. Unlike limited ablation analyses restricted to a single binary task, this study examines the impact of component removal across gender-based, age-based, and joint age–gender classification, thereby providing a comprehensive assessment under increasing task complexity.

##### Ablation design and experimental protocol

Starting from the full proposed framework, four core components were ablated independently:

(i) demographic-aware task stratification,
(ii) OptABC-based hyperparameter optimization,
(iii) CED-based structural preprocessing, and.
(iv) data augmentation.

Each ablated configuration modifies only one component at a time, ensuring that observed performance differences can be directly attributed to the removed or altered module. All experiments were conducted using the same five-fold cross-validation protocol and identical data splits to guarantee a fair and controlled comparison.

##### Quantitative impact of component removal

The quantitative results of the ablation study are summarized in Table 8, which reports classification accuracy across gender-based, age-based, and joint age–gender tasks.

**Table 8:** Ablation study evaluating the contribution of key components of the proposed framework across demographic-aware classification tasks, including gender-based, age-based, and joint age–gender classification. Each row reports classification accuracy after systematically removing or modifying individual components, such as preprocessing, data augmentation, and OptABC-based hyperparameter optimization. The results illustrate the relative impact of each component on overall performance and demonstrate their complementary roles in achieving robust classification.

Configuration	Gender-based accuracy (%)	Age-based accuracy (%)	Joint age–gender accuracy (%)
Full proposed framework	84.25	88.07	71.58
Without demographic stratification	80.62	82.14	—
Without OptABC optimization (fixed hyperparameters)	81.03	84.11	66.42
Without CED preprocessing	82.17	85.26	68.03
Without data augmentation	80.94	83.68	65.87

As shown in Table 8, the full proposed framework consistently achieves the highest performance across all classification settings. The reported values for the non-stratified configuration correspond to baseline binary ASD versus TD classification settings evaluated under the gender-oriented and age-oriented experimental protocols, respectively. This confirms that demographic-aware decomposition enables the model to learn more homogeneous and discriminative representations, which is critical for capturing ASD heterogeneity.

The removal of OptABC-based hyperparameter optimization results in the largest performance degradation, especially for the joint age–gender task. This observation highlights the importance of adaptive hyperparameter tuning when dealing with fine-grained multi-class problems and heterogeneous neuroimaging data. In contrast, fixed hyperparameter configurations limit the model’s ability to balance capacity and generalization across demographic subgroups.

Excluding CED-based preprocessing also leads to a measurable reduction in accuracy, indicating that explicit structural localization supports the learning of discriminative anatomical features from sMRI data. Similarly, removing data augmentation degrades performance and increases sensitivity to class imbalance, reflecting reduced robustness to inter-site and inter-subject variability.

##### Interpretation and methodological implications

The progressive performance degradation observed across ablated configurations demonstrates that the proposed framework is not a collection of independent heuristics, but rather a tightly integrated system in which each component plays a complementary role. Notably, the relative impact of each component becomes more pronounced as task complexity increases, with the joint age–gender classification representing the most challenging scenario.

These findings are consistent with the theoretical interpretation discussed earlier, where demographic-aware task decomposition reduces intra-class variance, and OptABC-based bi-level optimization enhances generalization under complex decision boundaries. The ablation study therefore provides empirical validation for both the methodological design and the theoretical motivation of the proposed framework.

Overall, the ablation analysis confirms that demographic stratification, adaptive optimization, structural preprocessing, and data augmentation jointly contribute to stable learning, improved robustness, and enhanced classification performance, thereby reinforcing the validity and necessity of the proposed design choices.

#### Mathematical perspective and theoretical interpretation

Although the primary contribution of this work lies in system design and empirical evaluation, the proposed framework can be formally interpreted within a mathematical modeling perspective. Specifically, the ASD classification problem addressed in this study can be viewed as a multi-class probabilistic learning problem defined over high-dimensional anatomical feature spaces derived from structural MRI data.

Let $\:\mathcal{X}\subset\:{\mathbb{R}}^{d}\:$ denote the space of structural brain representations extracted implicitly by the convolutional neural networks, and let $\:\mathcal{Y}=\{1,\dots\:,\mathrm{K}\}$ represent the set of demographic-aware class labels. The learning objective of each CNN model is to approximate a conditional probability mapping $f_{\theta } :{\mathcal{X}} \to {\mathcal{P}}\left({\mathcal{Y}} \right)$, parameterized by $\:\theta\:$, that minimizes empirical risk under cross-entropy loss. While this formulation is standard in supervised learning, its application to demographic-stratified neuroimaging classification introduces additional structural complexity due to heterogeneous class distributions and overlapping anatomical patterns.

From an optimization standpoint, the use of the Optimized Artificial Bee Colony (OptABC) algorithm introduces a metaheuristic search layer over the model parameter space. Unlike gradient-based optimization, OptABC performs population-based exploration over discrete and continuous hyperparameter domains, enabling adaptive control of model capacity. This can be interpreted as a bi-level optimization process, where the outer loop searches for optimal hyperparameters, and the inner loop performs gradient-based learning of network weights. Such bi-level formulations have been increasingly adopted in complex deep learning systems to balance expressiveness and generalization^54,55^.

Importantly, the demographic-aware modeling strategy implicitly introduces a structured partitioning of the label space, transforming a single binary decision boundary into multiple demographic-specific decision manifolds. This decomposition reduces intra-class variance within each subtask while increasing inter-class separability, particularly for age-based stratification. The observed performance differences across gender-based, age-based, and joint age–gender tasks can therefore be interpreted as a function of the geometric complexity of class manifolds in the learned feature space. Similar interpretations have been reported in recent neuroimaging studies addressing multi-task and stratified learning problems^32,33^.

The extended performance analysis presented in Figs. 8 and 9 further supports this interpretation. Smooth convergence behavior reflects stable optimization dynamics, while structured misclassification patterns indicate gradual transitions between neighboring demographic classes rather than random errors. These findings suggest that the learned representations preserve meaningful anatomical continuity, which is consistent with known neurodevelopmental processes.

Overall, while the proposed framework does not introduce a new mathematical theory, it contributes a mathematically grounded formulation of demographic-aware ASD classification, combining probabilistic learning, bi-level optimization, and structured label decomposition. This perspective elevates the discussion beyond empirical observation and provides a theoretical basis for interpreting the observed performance trends, limitations, and future extensions.

#### Benefits and research contribution of the proposed framework

The primary contribution of the proposed framework is its demographic-aware design, which moves beyond conventional binary ASD classification to explicitly model age- and gender-related heterogeneity. This approach reflects known clinical variability in ASD presentation and provides a richer analytical perspective on subgroup-specific neuroanatomical patterns. Combined with automated optimization via OptABC and moderate computational demands, the proposed framework emphasizes balanced performance, scalability, and clinical relevance rather than accuracy alone.

#### Discussion on fairness and limitations of SOTA comparison

Direct quantitative comparison with SOTA studies is inherently constrained by differences in imaging modality, cohort selection, preprocessing pipelines, and validation protocols. Accordingly, the presented comparison aims to contextualize the proposed framework within the broader research landscape rather than claim absolute superiority. Future work will focus on standardized benchmarking and evaluation across independent cohorts to further strengthen comparative conclusions.

### Limitations

Although the results presented in Table 3; Fig. 5 demonstrate strong performance, several limitations remain. First, the experiments were conducted using a single public dataset, and although ABIDE is multi-site and diverse, external validation on independent cohorts is necessary to confirm generalizability. Accordingly, the quantitative performance results reported in the present study should be interpreted as evidence of within-ABIDE generalization under the adopted experimental protocol rather than as confirmation of cross-cohort or cross-protocol generalizability. Second, the study focuses exclusively on structural MRI data; incorporating additional modalities such as functional MRI or diffusion imaging may further enhance diagnostic performance. Finally, while the proposed framework demonstrates strong classification accuracy, it has not yet been evaluated in real clinical workflows, where factors such as acquisition time, interpretability, and clinician interaction play critical roles.

Although the current study uses conservative geometric and noise-based augmentation suitable for sMRI classification, future work will explore more advanced augmentation strategies (e.g., intensity-based normalization augmentation or anatomically constrained transformations) and will evaluate their impact across sites and demographic strata. In addition, the proposed preprocessing strategy relies on Canny Edge Detection (CED) as a lightweight structural enhancement approach, which differs from widely adopted anatomical preprocessing frameworks such as FreeSurfer, SPM, and FSL. Although the ablation analysis suggests that CED contributes positively within the proposed framework, the absence of direct comparison with standardized sMRI preprocessing pipelines may limit cross-study comparability and should therefore be interpreted cautiously. In addition, future studies will investigate integrating standardized anatomical preprocessing tools with task-specific structural enhancement to further assess potential gains in robustness and reproducibility.

## Conclusion and future work

This study presented a demographic-aware deep learning framework for multi-class Autism Spectrum Disorder (ASD) classification using structural MRI (sMRI) data, with a particular focus on optimization-driven model design. Unlike conventional approaches that predominantly address ASD as a binary classification problem, the proposed framework formulates the task as a structured multi-class learning problem through three dedicated convolutional neural network (CNN) models targeting age-based, gender-based, and joint age–gender classification. This formulation enables a finer-grained mathematical representation of ASD-related neuroanatomical variability across demographic subgroups.

A key methodological contribution of this work lies in the integration of the Optimized Artificial Bee Colony (OptABC) algorithm for automatic hyperparameter optimization. By coupling metaheuristic optimization with deep learning, the framework systematically adapts network configurations to the specific characteristics of each classification task. This optimization strategy reduces reliance on manual tuning and enhances model efficiency and generalization. In addition, the use of a structured preprocessing pipeline, incorporating edge-based feature enhancement and controlled data augmentation, contributes to improved learning stability and robustness. Experimental evaluation using five-fold cross-validation on the multi-site ABIDE dataset demonstrates that the optimized models achieve competitive performance relative to widely used pre-trained CNN architectures, while extending analysis beyond traditional binary classification schemes.

The experimental results further indicate that incorporating demographic constraints—particularly age stratification—improves discriminative performance in ASD classification from sMRI data. These findings suggest that demographic-aware modeling, when combined with optimization-based deep learning, can capture meaningful structural variations associated with ASD and provide a more informative representation of disorder heterogeneity. From a computational perspective, the proposed framework offers favorable scalability and efficiency, making it suitable for large-scale neuroimaging analysis and optimization-driven learning scenarios.

Although the primary contribution of this work is methodological, the framework also demonstrates potential for practical deployment in decision-support contexts. The use of optimized 2D CNN architectures enables relatively low inference complexity, which is advantageous for large datasets and time-sensitive applications. Rather than serving as a standalone diagnostic tool, the proposed system is better positioned as a supportive analytical component that can assist clinicians by providing demographic-aware risk assessments derived from routine MRI data. The feasibility of real-time or near real-time deployment, however, remains an open research question and was not explicitly addressed in the current study.

Several limitations should be acknowledged. First, despite the heterogeneity of the ABIDE dataset, reliance on a single public dataset constrains conclusions regarding external generalizability. Second, variations in MRI acquisition protocols across sites may introduce confounding effects that are not explicitly modeled. Finally, the current framework is limited to structural MRI data and does not incorporate interpretability mechanisms, which are increasingly important for trust and usability in applied AI systems.

Future work will extend this research along multiple directions. External validation on independent neuroimaging cohorts, such as EU-AIMS LEAP, NDAR, and other large-scale ASD repositories, will be conducted to further assess robustness, reproducibility, and generalizability across diverse acquisition protocols and demographic distributions. Explainable AI techniques, such as gradient-based attribution and relevance propagation methods, will be integrated to improve interpretability and provide insight into the neuroanatomical features driving classification decisions. In addition, multimodal data fusion strategies combining structural, functional, and diffusion imaging will be explored to enhance representational richness. From an optimization and systems perspective, federated learning and longitudinal modeling will be investigated to support privacy-preserving, multi-institutional training and to capture developmental dynamics associated with ASD.

Overall, this work contributes an optimization-centric deep learning framework that advances multi-class, demographic-aware modeling in neuroimaging applications. While further validation and system-level refinement are necessary, the proposed approach provides a solid methodological foundation for future research at the intersection of artificial intelligence, machine learning, and optimization.

## Supplementary Information

Below is the link to the electronic supplementary material.

Supplementary Material 1

Supplementary Material 2

Supplementary Material 3

Supplementary Material 4

## Data Availability

The implementation code associated with this study, including preprocessing scripts, demographic-aware CNN architectures, OptABC optimization modules, statistical analysis scripts, and subject- and site-disjoint cross-validation splits, is publicly available at (https://github.com/mohammedaly-tech/Demographic-Aware-ASD-Classification).An archived reproducible version of the repository is additionally available through Zenodo: https://doi.org/10.5281/zenodo.20419300. The datasets analyzed during the current study are available in the Autism Brain Imaging Data Exchange (ABIDE) repository: https://fcon_1000.projects.nitrc.org/indi/abide/abide_I.html.

## References

[1] Rapin, I. & Tuchman, R. F. What is new in autism? Curr. *Opin. Neurol.* **21** (2), 143–149 (2008). 10.1097/WCO.0b013e3282f49579 18317271

[2] Mueller, S., Keeser, D., Reiser, M. F., Teipel, S. & Meindl, T. Functional and structural MR imaging in neuropsychiatric disorders, part 2: application in schizophrenia and autism. *AJNR Am. J. Neuroradiol.* **33**, 2033–2037 (2012). 22173749 10.3174/ajnr.A2800 PMC7965600

[3] Plitt, M., Barnes, K. A. & Martin, A. Functional connectivity classification of autism identifies highly predictive brain features but falls short of biomarker standards. *NeuroImage Clin.* **7**, 359–366 (2015). 25685703 10.1016/j.nicl.2014.12.013 PMC4309950

[4] Yang, X., Zhang, N. & Schrader, P. A study of brain networks for autism spectrum disorder classification using resting-state functional connectivity. *Mach. Learn. Appl.* **8**, 100290 (2022).

[5] Yu, Z. et al. Epileptic seizure prediction using deep neural networks via transfer learning and multi-feature fusion. *Int. J. Neural Syst.* **32**, 2250032 (2022). 35695914 10.1142/S0129065722500320

[6] Thangavel, P. et al. Time-frequency decomposition of scalp electroencephalograms improves deep learning-based epilepsy diagnosis. *Int. J. Neural Syst.* **31**, 2150032 (2021). 34278972 10.1142/S0129065721500325 PMC9340811

[7] Ardakani, H. A., Taghizadeh, M. & Shayegh, F. Diagnosis of autism disorder based on deep network trained by augmented EEG signals. *Int. J. Neural Syst.* **32**, 2250046 (2022). 35997585 10.1142/S0129065722500460

[8] Akhavan Aghdam, M., Sharifi, A. & Pedram, M. M. Combination of rs-fMRI and sMRI data to discriminate autism spectrum disorders in young children using deep belief network. *J. Digit. Imaging*. **31**, 895–903 (2018). 29736781 10.1007/s10278-018-0093-8 PMC6261184

[9] Ali, M. T. et al. Autism classification using sMRI: A recursive features selection based on sampling from multi-level high dimensional spaces. In *Proc. IEEE 18th Int. Symp. Biomed. Imaging (ISBI)*, 267–270 https://doi.org/10.1109/ISBI48211.2021.9433973 (2021).

[10] Subbaraju, V., Sundaram, S., Narasimhan, S. & Suresh, M. B. Accurate detection of autism spectrum disorder from structural MRI using extended metacognitive radial basis function network. *Expert Syst. Appl.* **42** (22), 8775–8790 (2015).

[11] Kong, Y. et al. Classification of autism spectrum disorder by combining brain connectivity and deep neural network classifier. *Neurocomputing* **324**, 63–68 (2019).

[12] Tang, Y. et al. Multi-site diagnostic classification of autism spectrum disorder using adversarial deep learning on resting-state fMRI. *Biomed. Signal Process. Control* **85**, 104892 (2023).

[13] Khudhur, D. D. & Khudhur, S. D. The classification of autism spectrum disorder by machine learning methods on multiple datasets for four age groups. *Measurement: Sensors* **27**, 100774 (2023).

[14] Gorriz, J. M. et al. A machine learning approach to reveal the neurophenotypes of autisms. *Int. J. Neural Syst.*10.1142/S0129065718500582 (2019). 30782022 10.1142/S0129065718500582

[15] Khan, K. & Katarya, R. M. C. B. E. R. T. A multi-modal framework for the diagnosis of autism spectrum disorder. *Biol. Psychol.* **194**, 108976 (2025). 39722324 10.1016/j.biopsycho.2024.108976

[16] Khan, K. & Katarya, R. WS-BiTM: Integrating White Shark Optimization with Bi-LSTM for enhanced autism spectrum disorder diagnosis. *J. Neurosci. Methods*. **413**, 110319 (2025). 39521353 10.1016/j.jneumeth.2024.110319

[17] Khan, K. & Katarya, R. AFF-BPL: An adaptive feature fusion technique for the diagnosis of autism spectrum disorder using Bat-PSO-LSTM based framework. *J. Comput. Sci.* **83**, 102447 (2024).

[18] Khan, K. & Katarya, R. Machine learning techniques for autism spectrum disorder: Current trends and future directions. In *Proc. Int. Conf. Innov. Trends Inf. Technol. (ICITIIT)*, 1–7 (2023).

[19] Jha, A., Khan, K. & Katarya, R. Diagnosis support model for autism spectrum disorder using neuroimaging data and Xception. In *Proc. Int. Conf. Electr. Electron. Commun. Comput. (ELEXCOM)*, 1–6 (2023).

[20] Sethi, A., Khan, K., Katarya, R. & Yingthawornsuk, T. Empirical evaluation of machine learning techniques for autism spectrum disorder. In *Proc. Int. Electr. Eng. Congr. (iEECON)*, 1–5 (2024).

[21] Khan, K., Katarya, R. & S/SD-ASD Self-supervised and self-distillation learning approach for classifying autism spectrum disorder in children using facial images. *Eng. Anal. Bound. Elem.* **179**, 106382 (2025).

[22] Autism Brain Imaging Data Exchange (ABIDE). 15, http://fcon_1000.projects.nitrc.org/indi/abide/ (2023).

[23] Di Martino, A. et al. The autism brain imaging data exchange: towards a large-scale evaluation of the intrinsic brain architecture in autism. *Mol. Psychiatry* **19**(6), 659–667. 10.1038/mp.2013.78 (2014). 23774715 10.1038/mp.2013.78 PMC4162310

[24] Garcea, F., Serra, A., Lamberti, F. & Morra, L. Data augmentation for medical imaging: A systematic literature review. *Comput. Biol. Med.* **152**, 106391 (2023). 36549032 10.1016/j.compbiomed.2022.106391

[25] Havaei, M. et al. Brain tumor segmentation with deep neural networks. *Med. Image Anal.* **35**, 18–31. 10.1016/j.media.2016.05.004 (2017). 27310171 10.1016/j.media.2016.05.004

[26] Shorten, C. & Khoshgoftaar, T. M. A survey on image data augmentation for deep learning. *J. Big Data*. **6** (1), 1–48. 10.1186/s40537-019-0197-0 (2019). 10.1186/s40537-021-00492-0 PMC8287113 34306963

[27] Perez, L. & Wang, J. The effectiveness of data augmentation in image classification using deep learning. *arXiv preprint *https://arXiv.org/abs/171204621 (2017).

[28] Sujana, D. S. & Augustine, D. P. The effect of spatial and intensity level augmentation of structural magnetic resonance images on autism diagnosis model. *Asian J. Psychiatry* 104830 (2026). 10.1016/j.ajp.2026.104830 41499905

[29] Litjens, G. et al. A survey on deep learning in medical image analysis. *Med. Image Anal.* **42**, 60–88 (2017). 28778026 10.1016/j.media.2017.07.005

[30] Bauer, S., Wiest, R., Nolte, L. P. & Reyes, M. A survey of MRI-based medical image analysis for brain tumor studies. *Phys. Med. Biol.* **58** (13), R97–R129. 10.1088/0031-9155/58/13/R97 (2013). 23743802 10.1088/0031-9155/58/13/R97

[31] Zahedi, L., Mohammadi, F. G. & Amini, M. H. OptABC: An optimal hyperparameter tuning approach for machine learning algorithms. In *Proc. IEEE Int. Conf. Mach. Learn. Appl. (ICMLA)*, 1138–1145 (2021).

[32] Garcia, M. & Kelly, C. 3D CNN for neuropsychiatry: Predicting autism with interpretable deep learning applied to minimally preprocessed structural MRI data. *PLoS One*. **19** (10), e0276832 (2024). 39432512 10.1371/journal.pone.0276832 PMC11493284

[33] Nogay, H. S. & Adeli, H. Multiple classification of brain MRI autism spectrum disorder by age and gender using deep learning. *J. Med. Syst.* **48** (1), 15 (2024). 38252192 10.1007/s10916-023-02032-0 PMC10803393

[34] Mishra, M. & Pati, U. C. A classification framework for autism spectrum disorder detection using sMRI: Optimizer-based ensemble of deep convolution neural network with on-the-fly data augmentation. *Biomed. Signal. Process. Control*. **84**, 104686 (2023).

[35] Saponaro, S. et al. Deep learning based joint fusion approach to exploit anatomical and functional brain information in autism spectrum disorders. *Brain Inf.* **11** (1), 2 (2024). 10.1186/s40708-023-00217-4 PMC10776521 38194126

[36] Vidya, S. et al. Identification of critical brain regions for autism diagnosis from fMRI data using explainable AI: An observational analysis of the ABIDE dataset. *EClinicalMedicine*10.1016/j.eclinm.2025.103452 (2025). 41181843 10.1016/j.eclinm.2025.103452 PMC12573454

[37] Esteva, A. et al. A guide to deep learning in healthcare. *Nat. Med.* **25** (1), 24–29 (2019). 30617335 10.1038/s41591-018-0316-z

[38] Elsayed, E. K., Salem, D. R. & Aly, M. A fast quantum particle swarm optimization algorithm for image denoising problem. *International Journal of Intelligent Engineering and Systems*10.22266/ijies2020.0229.10 (2020).

[39] Elsayed, E. & Aly, M. Hybrid between ontology and quantum particle swarm optimization for segmenting noisy plant disease image. *Int. J. Intell. Eng. Syst.* **12** (5), 299–311 (2019).

[40] Aly, M. & Alotaibi, N. S. A novel deep learning model to detect COVID-19 based on wavelet features extracted from Mel-scale spectrogram of patients’ cough and breathing sounds. *Informatics in Medicine Unlocked* **32**, 101049 (2022). 35989705 10.1016/j.imu.2022.101049 PMC9375256

[41] Aly, M. & Alotaibi, N. S. A new model to detect COVID-19 coughing and breathing sound symptoms classification from CQT and Mel spectrogram image representation using deep learning. *International Journal of Advanced Computer Science and Applications*10.14569/ijacsa.2022.0130869 (2022).

[42] Aly, M. & Alotaibi, A. S. Molecular property prediction of modified Gedunin using machine learning. *Molecules* **28** (3), 1125 (2023). 36770791 10.3390/molecules28031125 PMC9921289

[43] Hjazi, A. et al. Optimization of removal of sulfonamide antibiotics by magnetic nanocomposite from water samples using central composite design. *Water Resour. Ind.*10.1016/j.wri.2023.100229 (2023).

[44] Aly, M. & Alotaibi, A. S. Emu-net: Automatic brain tumor segmentation and classification using efficient modified U-Net. *Comput. Mater. Continua*. **77** (1), 557–582 (2023).

[45] Aly, M., Ghallab, A. & Fathi, I. S. Enhancing facial expression recognition system in online learning context using efficient deep learning model. *IEEE Access.* **11**, 121419–121433. 10.1109/ACCESS.2023.3325407 (2023).

[46] Behiry, M. H. & Aly, M. Cyberattack detection in wireless sensor networks using a hybrid feature reduction technique with AI and machine learning methods. *J. Big Data*. **11**, 16. 10.1186/s40537-023-00870-w (2024).

[47] Aly, M., Ghallab, A. & Fathi, I. S. Tumor ViT-GRU-XAI: Advanced Brain Tumor Diagnosis Framework: Vision Transformer and GRU Integration for Improved MRI Analysis: A Case Study of Egypt. *IEEE Access*10.1109/access.2024.3513235 (2024).

[48] Aly, M. Revolutionizing online education: Advanced facial expression recognition for real-time student progress tracking via deep learning model. *Multimed. Tools Appl.*10.1007/s11042-024-19392-5 (2024).

[49] Aly, M. Weakly-supervised thyroid ultrasound segmentation: Leveraging multi-scale consistency, contextual features, and bounding box supervision for accurate target delineation. *Comput. Biol. Med.* **186**, 109669 (2025). 39809086 10.1016/j.compbiomed.2025.109669

[50] Aly, M. & Alotaibi, A. S. Hybrid Butterfly-Grey Wolf Optimization (HB-GWO): A novel metaheuristic approach for feature selection in high-dimensional data. *Stat. Optim. Inf. Comput.* **13** (6), 2575–2600 (2025).

[51] Aly, M. & Fathi, I. S. Recognizing American Sign Language gestures efficiently and accurately using a hybrid transformer model. *Sci. Rep.* **15** (1), 1–27 (2025). 40550837 10.1038/s41598-025-06344-8 PMC12185765

[52] Fathi, I. S., Ardah, H., Hassan, G. & Aly, M. Protecting IoT networks through AI-based solutions and fractional Tchebichef moments. *Fractal Fract.* **9** (7), 427. 10.3390/fractalfract9070427 (2025).

[53] Aly, M. & Behiry, M. H. Enhancing anomaly detection in IoT-driven factories using logistic boosting, random forest, and SVM: A comparative machine learning approach. *Sci. Rep.* **15**, 23694. 10.1038/s41598-025-08436-x (2025). 40610511 10.1038/s41598-025-08436-x PMC12229705

[54] Franceschi, L., Frasconi, P., Salzo, S., Grazzi, R. & Pontil, M. Bilevel programming for hyperparameter optimization and meta-learning. In *Proc. Int. Conf. Mach. Learn.*, 1568–1577 (2018).

[55] Feurer, M. & Hutter, F. Hyperparameter optimization. In *Automated Machine Learning: Methods, Systems, Challenges* 3–33 (Springer, 2019).

