# Supplementary material for: Metaheuristic hyperparameter optimization of deep neural networks for demographic-aware autism spectrum disorder classification
Source: Sci Rep. 2026 Jun 29;16:19793. doi: 10.1038/s41598-026-58789-0 (PMC13316114; doi:10.1038/s41598-026-58789-0)
Supplement: Supplementary file 1 — Supplementary Material 1 [file 41598_2026_58789_MOESM1_ESM.docx]

**Supplementary Material A: Extended Implementation Details**

This supplementary document provides additional implementation details to support reproducibility of the proposed framework, while maintaining a concise and focused presentation in the main manuscript. In line with contemporary editorial practices in high-impact medical imaging journals, standard deep learning components and low-level configuration details are consolidated here to avoid redundancy in the primary text.

**A.1 Network Output Layer and Loss Function**

All CNN models employed in this study utilize a softmax activation function at the output layer to model multi-class probability distributions. The softmax function converts raw network outputs into normalized class probabilities, enabling probabilistic interpretation of classification decisions. Given its widespread adoption in multi-class deep learning tasks, the formulation is provided here for completeness.

$$\sigma\left( z_{i} \right)=\frac{e^{z_{i}}}{\sum_{j=1}^{K} e^{z_{j}}}$$

where $z_{i}$denotes the output score for class $i$, and $K$ is the number of target classes.

Model optimization is performed using the categorical cross-entropy loss function, which penalizes incorrect predictions proportionally to their confidence. This loss function is standard for supervised multi-class classification and is widely adopted in medical image analysis.

$$\mathcal{L=-}\sum_{i=1}^{K} y_{i} log(\hat{y_{i}})$$

where $y_{i}$and $\hat{y_{i}}$denote the ground-truth and predicted probabilities, respectively.

**A.2 Evaluation Metrics**

To ensure objective and interpretable performance evaluation, standard classification metrics are employed. These metrics are defined here for completeness, as they are well established in the literature and not the focus of methodological innovation in this study.

**Supplementary Table A1. Definitions of Evaluation Metrics**

| **Metric** | **Definition** |
| --- | --- |
| Accuracy | Proportion of correctly classified samples among all samples |
| Precision | Ratio of true positive predictions to all positive predictions |
| Recall (Sensitivity) | Ratio of true positive predictions to all actual positive samples |
| F1-score | Harmonic mean of precision and recall |

These metrics were computed under a five-fold cross-validation protocol and averaged across folds to ensure robustness against data partitioning bias.

**A.3 Extended Preprocessing Operations**

Beyond the task-specific preprocessing steps discussed in the main manuscript, several routine operations were applied to ensure data consistency across sites. These steps are common in sMRI-based deep learning studies and are summarized here.

**Supplementary Table A2. Summary of Routine Preprocessing Operations**

| **Step** | **Description** |
| --- | --- |
| Image resizing | Standardization to a fixed spatial resolution |
| Intensity normalization | Min–max normalization to reduce scanner-dependent intensity variation |
| Background removal | Elimination of non-brain regions to reduce redundancy |
| Quality filtering | Automated removal of low-contrast or blurred images |

These operations are widely used in neuroimaging pipelines and serve as preparatory steps rather than sources of methodological novelty.

**A.4 Hyperparameter Search Space**

Hyperparameter tuning was performed using the Optimized Artificial Bee Colony (OptABC) algorithm. Rather than reporting exhaustive parameter-level detail in the main manuscript, the search space explored by OptABC is documented here for reproducibility.

**Supplementary Table A3. Hyperparameter Search Space for CNN Optimization**

| **Parameter** | **Search Range** |
| --- | --- |
| Number of convolutional layers | 3 – 6 |
| Number of filters per layer | 16 – 64 |
| Filter size | 2×2 – 5×5 |
| Stride | 1 – 2 |
| Padding | Valid / Same |
| Learning rate | 1e−4 – 3e−4 |
| Mini-batch size | 16 – 32 |
| Momentum | 0.7 – 0.9 |
| L2 regularization | 1e−4 |

The final selected configurations for each demographic-specific model are reported concisely in the main manuscript, while this table documents the optimization boundaries.

**A.5 Data Augmentation Parameters**

To mitigate class imbalance and improve generalization, controlled data augmentation was applied during training. These operations were selected to preserve anatomical integrity while increasing sample diversity.

**Supplementary Table A4. Data Augmentation Settings**

| **Augmentation Type** | **Parameter** |
| --- | --- |
| Horizontal flip | Probability = 0.5 |
| Rotation | ±90°, ±180° |
| Gaussian noise | Variance = 0.01 |
| Scaling | ±10% |

Augmentation was applied only to the training set within each cross-validation fold to avoid data leakage.

**A.6 Reproducibility and Implementation Notes**

All experiments were conducted under a fixed random seed to ensure reproducibility. Training and evaluation were performed using consistent data splits across all models, and no subject appeared in both training and testing sets within the same fold. The implementation follows widely accepted deep learning practices, with all deviations from standard pipelines explicitly described in the main manuscript.
