# Supplementary material for: Metaheuristic hyperparameter optimization of deep neural networks for demographic-aware autism spectrum disorder classification
Source: Sci Rep. 2026 Jun 29;16:19793. doi: 10.1038/s41598-026-58789-0 (PMC13316114; doi:10.1038/s41598-026-58789-0)
Supplement: Supplementary file 2 — Supplementary Material 2 [file 41598_2026_58789_MOESM2_ESM.docx]

**Supplementary Material A: Preprocessing and Hyperparameter Configuration**

**S1. Data Preprocessing Details**

**S1.1 Image Resizing and Normalization**

All structural MRI slices were resized to a fixed spatial resolution to ensure uniform input dimensions across subjects and acquisition sites. Intensity normalization was applied using min–max scaling to map voxel intensities to a standardized range, improving numerical stability during training and reducing scanner-dependent intensity variability. This normalization strategy is widely used in sMRI-based deep learning studies and avoids introducing assumptions specific to functional imaging.

**S1.2 Data Augmentation Strategy**

Data augmentation was employed exclusively on the training set to enhance robustness and reduce class imbalance. The following anatomically plausible transformations were applied:

- Horizontal flipping
- Rotation (±90° and 180°)
- Mild Gaussian noise injection (variance = 0.01)

These transformations preserve neuroanatomical integrity while increasing data diversity. Augmentation was deliberately constrained to avoid unrealistic deformations that could bias structural interpretation. No augmentation was applied to validation or test samples.

**S2. Hyperparameter Configuration and Rationale**

**S2.1 Optimization Settings**

All CNN models were trained using mini-batch gradient descent with adaptive learning rates. Batch size was fixed at 32, which represents a balance between convergence stability and GPU memory efficiency, and is commonly adopted in neuroimaging deep learning studies. Smaller batch sizes were observed to increase gradient variance, while larger batches did not yield consistent performance gains.

**S2.2 Learning Rate and Regularization**

Initial learning rates were selected within the range of 10⁻⁴ to 10⁻³, consistent with best practices for CNN training on medical imaging datasets. These values provide stable convergence without oscillatory behavior. L2 regularization was applied to convolutional layers to mitigate overfitting, particularly given the high dimensionality of sMRI data.

**S2.3 Hyperparameter Selection Strategy**

Rather than relying on arbitrary manual tuning, hyperparameters were optimized using the Optimized Artificial Bee Colony (OptABC) algorithm. OptABC explores the hyperparameter search space through a population-based metaheuristic strategy, balancing exploration and exploitation. This approach reduces dependence on heuristic trial-and-error selection and allows model configurations to adapt to different classification tasks (gender, age, and joint stratification).

The final hyperparameter values represent stable solutions identified by OptABC across multiple training runs rather than single optimal points.

**S2.4 Summary Table**

**Table S1. Summary of Training Hyperparameters**

| **Parameter** | **Value Range** | **Selection Rationale** |
| --- | --- | --- |
| Batch size | 32 | Stability vs. memory efficiency |
| Learning rate | 1e-4 – 3e-4 | Stable convergence |
| Optimizer | SGD (adaptive) | Robust training |
| Regularization | L2 (1e-4) | Overfitting control |
| Augmentation | Geometric + noise | Generalization |

**S3. Hyperparameter Selection Rationale**

**S3.1 Batch Size Selection**

A batch size of 32 was selected as it provides a balance between gradient stability and computational efficiency. Smaller batch sizes were observed to introduce higher variance in gradient updates, while larger batches did not yield consistent performance improvements given memory constraints and dataset heterogeneity. This choice is consistent with common practices in deep learning for medical image analysis.

**S3.2 Learning Rate Selection**

Learning rates in the range of 10⁻⁴ to 10⁻³ were explored by the OptABC algorithm. These values enable stable convergence without oscillatory behavior and are well-suited for training CNNs on high-dimensional structural MRI data. The final learning rates reported represent stable solutions identified across multiple optimization iterations rather than single trial outcomes.

**S3.3 Regularization and Dropout Strategy**

L2 regularization was employed to reduce overfitting by constraining model complexity, particularly given the limited availability of labeled neuroimaging data. Dropout layers were applied selectively in fully connected layers to improve generalization while avoiding disruption of spatial feature learning in convolutional layers.
