# Supplementary material for: Metaheuristic hyperparameter optimization of deep neural networks for demographic-aware autism spectrum disorder classification
Source: Sci Rep. 2026 Jun 29;16:19793. doi: 10.1038/s41598-026-58789-0 (PMC13316114; doi:10.1038/s41598-026-58789-0)
Supplement: Supplementary file 3 — Supplementary Material 3 [file 41598_2026_58789_MOESM3_ESM.docx]

**Supplementary Material for:**

**Autism Classification Using Structural MRI with Optimized Convolutional Neural Networks Based on Age and Gender Stratification**

**S1. Purpose of the Supplementary Material**

This Supplementary Material provides implementation-specific details that support the reproducibility of the proposed framework while maintaining clarity and conciseness in the main manuscript. In response to reviewer feedback, detailed hyperparameter configurations and CNN architectural specifications have been relocated from the main text to this document, as they are not essential for conceptual understanding but are important for technical replication.

**S2. CNN Hyperparameter Optimization Using OptABC**

All Convolutional Neural Network (CNN) models in this study were optimized using the Optimized Artificial Bee Colony (OptABC) algorithm. OptABC automatically selected the optimal architectural and training parameters for each classification task based on cross-validation performance.

The following parameters were optimized:

- Number of convolutional layers
- Number of max-pooling layers
- Number of fully connected layers
- Number and size of convolutional filters
- Padding and stride configurations
- Learning rate and momentum
- Mini-batch size
- L2 regularization strength

The activation function was fixed to ReLU across all models to ensure training stability.

**S3. Hyperparameter Configuration for Gender-Based Classification (Method 1)**

Table S1 presents the optimized hyperparameters for the CNN model trained for gender-based ASD classification (Method 1).

**Table S1. Optimized CNN Hyperparameters for Method 1 (Gender-Based Classification)**

| **Hyperparameter** | **Value** |
| --- | --- |
| Number of convolutional layers | 6 |
| Number of max-pooling layers | 6 |
| Number of fully connected layers | 2 |
| Filter configuration | [48, 48, 24, 24, 24, 24, 24, 24, 16, 16, 16, 16, 16, 16] |
| Filter sizes | [3, 4, 3, 3, 5, 4, 5, 5, 3, 3, 3, 3, 5, 4] |
| Padding | [0, 0, 1, 0, 1, 0, 0, 0, 1, 0, 0, 0, 0, 1] |
| Stride | [2, 1, 2, 1, 2, 1, 1, 1, 1, 2, 1, 1, 2, 1] |
| Learning rate | 0.0002 |
| Momentum | 0.70 |
| Mini-batch size | 32 |
| L2 regularization | 0.0001 |
| Activation function | ReLU |

**S4. Hyperparameter Configuration for Age-Based Classification (Method 2)**

Table S2 summarizes the optimized hyperparameters for the CNN model trained for age-group ASD classification (Method 2).

**Table S2. Optimized CNN Hyperparameters for Method 2 (Age-Based Classification)**

| **Hyperparameter** | **Value** |
| --- | --- |
| Number of convolutional layers | 6 |
| Number of max-pooling layers | 6 |
| Number of fully connected layers | 2 |
| Filter configuration | [48, 48, 32, 32, 24, 24, 24, 24, 16, 16, 16, 16] |
| Filter sizes | [4, 3, 3, 3, 5, 4, 3, 5, 4, 5, 5, 4] |
| Padding | [0, 0, 1, 0, 0, 1, 0, 0, 0, 0, 0, 0] |
| Stride | [1, 1, 1, 2, 1, 1, 1, 1, 2, 1, 2, 1] |
| Learning rate | 0.0003 |
| Momentum | 0.70 |
| Mini-batch size | 32 |
| L2 regularization | 0.0001 |
| Activation function | ReLU |

**S5. Hyperparameter Configuration for Joint Gender–Age Classification (Method 3)**

Table S3 presents the optimized hyperparameters for the CNN model used in joint gender–age (octal) classification (Method 3).

**Table S3. Optimized CNN Hyperparameters for Method 3 (Joint Gender–Age Classification)**

| **Hyperparameter** | **Value** |
| --- | --- |
| Number of convolutional layers | 3 |
| Number of max-pooling layers | 3 |
| Number of fully connected layers | 2 |
| Filter configuration | [48, 48, 32, 32] |
| Filter sizes | [2, 3, 4, 3] |
| Padding | [0, same, 0, 1] |
| Stride | [2, 2, 1, 2] |
| Learning rate | 0.0001 |
| Momentum | 0.90 |
| Mini-batch size | 32 |
| L2 regularization | 0.0001 |
| Activation function | ReLU |

**S6. CNN Training Protocol**

All CNN models were trained from scratch using their respective demographic-specific datasets. A 70/30 train–test split was employed, and five-fold cross-validation was used to ensure statistical robustness and reduce variance across data partitions. Early stopping was applied based on validation performance to mitigate overfitting.

**S7. Reproducibility Statement**

All preprocessing steps, model architectures, and training procedures described in the main manuscript and this Supplementary Material are sufficient to reproduce the reported results. Source code and trained model weights are available from the corresponding author upon reasonable request.
